# Tailoring nanoscopic confines to maximize catalytic activity of hydronium ions

**DOI:** 10.1038/ncomms15442

**Published:** 2017-05-25

**Authors:** Hui Shi, Sebastian Eckstein, Aleksei Vjunov, Donald M. Camaioni, Johannes A. Lercher

**Affiliations:** 1 Institute for Integrated Catalysis, Pacific Northwest National Laboratory, P.O. Box 999, Richland, Washington 99352, USA; 2 Department of Chemistry and Catalysis Research Center, TU München, Lichtenbergstrasse 4, 85748 Garching, Germany

## Abstract

Acid catalysis by hydronium ions is ubiquitous in aqueous-phase organic reactions. Here we show that hydronium ion catalysis, exemplified by intramolecular dehydration of cyclohexanol, is markedly influenced by steric constraints, yielding turnover rates that increase by up to two orders of magnitude in tight confines relative to an aqueous solution of a Brønsted acid. The higher activities in zeolites BEA and FAU than in water are caused by more positive activation entropies that more than offset higher activation enthalpies. The higher activity in zeolite MFI with pores smaller than BEA and FAU is caused by a lower activation enthalpy in the tighter confines that more than offsets a less positive activation entropy. Molecularly sized pores significantly enhance the association between hydronium ions and alcohols in a steric environment resembling the constraints in pockets of enzymes stabilizing active sites.

## Abstract (web summary)

The rates of acid-catalysed reactions vary in constrained environments. Here the authors show that molecularly sized pores greatly promote aqueous phase alcohol dehydration by enhancing the association between substrate and hydronium ions, and even by lowering the free energy barrier.

Understanding and maximizing the rates of acid-catalysed dehydration of alcohols is key to many synthetic strategies^1^,^2^,^3^,^4^,^5^,^6^. For reactions catalysed at the gas–solid interface, the catalytic consequences of acid strength^7^,^8^,^9^,^10^, charge reorganization^9^,^11^ and spatial constraints^8^,^10^,^12^ have been studied in detail. At the solid–liquid interface, however, the parameters controlling acid–base catalysis are hardly understood. Extrapolation from gas-phase reactions is challenging, as the organization in condensed phase has profound consequences^13^ for the interaction of the reacting substrates with the catalyst, the nature of the acid/base active sites as well as the reaction pathway.

In the presence of high concentrations of water, nearly complete proton transfer occurs from acidic hydroxyls at the solid surface to a cluster of water molecules^14^,^15^,^16^,^17^,^18^,^19^,^20^,^21^, making hydrated hydronium ions (for example, (H_2_O)_n_·H_3_O^+^) the prevalent form of Brønsted acid sites (BAS). However, the structure–function relations of hydronium ions generated at solid surfaces are complex^3^,^22^. We showed previously^23^ that the catalytic activity of hydronium ions for aqueous-phase cyclohexanol dehydration is more than an order of magnitude higher in HBEA zeolite than in the acidic solutions. Understanding the causes and consequences of such rate enhancement induced by confined nanoenvironments is the key to designing novel catalysts that maximize acid-catalysed rates in condensed phase by utilizing inorganic and organic nanoenvironments.

We report here on the fundamentals of the impact of steric confines on hydronium ions and the kinetic and thermodynamic consequences for catalysis, using aqueous-phase dehydration of cyclohexanol as a prototypical example. We show that properly sized confines can be exploited to both maximize the substrate–hydronium ion association and lower the intrinsic free energy of activation for alcohol dehydration. The rate of hydronium ion-catalysed elimination reactions can vary by up to two orders of magnitude with changing size of the confines, relative to aqueous-phase acid solutions, pointing to the possibility of designing catalysts that can achieve enzyme-like rates for acid-catalysed reactions under mild conditions.

## Results

### Dehydration kinetics over homogeneous and solid acids

**Figure 1:**
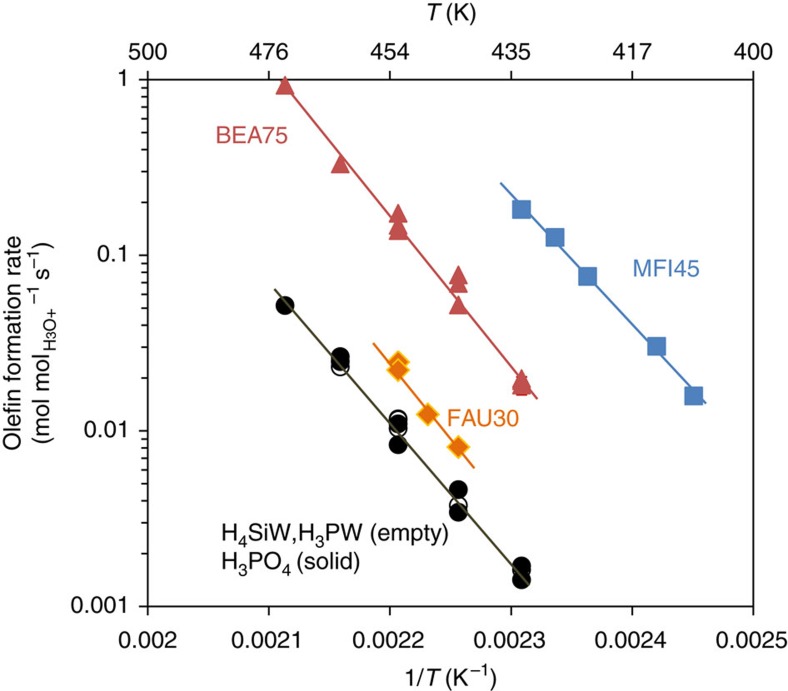
Measured turnover frequencies of cyclohexene formation in aqueous-phase dehydration of cyclohexanol on different acid catalysts. For zeolites, the Si/Al ratio is denoted as the number following the framework-type code. H_4_SiW and H_3_PW stand for tungstosilicic and phosphotungstic acids. Rates were determined at conversions <10%. Solid lines are fits to the Arrhenius equation (note that the slopes are not identical).

Figure 1 compiles the formation rates of cyclohexene from cyclohexanol dehydration over different acids. All homogeneous acids (H_3_PW_12_O_40_, H_4_SiW_12_O_40_, HClO_4_ and H_3_PO_4_, <0.1 M) showed identical rates when normalized to the concentration of hydronium ions (abbreviated as H_3_O^+^_aq_), that is, all these acids had identical turnover frequencies. The H_3_O^+^_aq_ concentration in pure liquid water was too low to catalyse the reaction at detectable rates at these temperatures.

The initial rates on solid acids (physicochemical characteristics of solid acids shown in Supplementary Table 1) have been normalized to the concentrations of BAS determined from titration experiments using aqueous solutions of pyridine or 2,6-dimethylpyridine (see Methods), with the latter base known to selectively titrate BAS^24^,^25^. The similar decrease in rates with increasing titrant uptakes for the two bases (Supplementary Fig. 1) provides evidence that hydronium ions are the active site and that Lewis acid sites (those that have not been converted to BAS in aqueous phase) do not contribute to aqueous-phase dehydration of cyclohexanol.

The turnover frequencies (TOFs) decreased with increasing pore dimensions (MFI: 0.51 nm × 0.55 nm and 0.53 nm × 0.56 nm, BEA: 0.66 nm × 0.67 nm and 0.56 nm × 0.58 nm, FAU: 0.74 nm × 0.74 nm^26^). As the highest activity was observed with MFI having smaller pores than those of BEA and FAU, we rule out that differences are caused by consequences of pore diffusion constraints. While Fig. 1 illustrates TOFs measured at a certain Si/Al ratio for each framework structure, TOFs were not much affected by the concentration of hydronium ions (Supplementary Note 1). A small-pore zeolite, chabazite (CHA) showed, however, a very low activity (Supplementary Fig. 2). As the size of cyclohexanol is much larger than the pore opening of CHA, the measured low rates are considered to arise predominantly from sites at the external crystalline surface of CHA. These results further indicate that hydronium ions are not accessible in the chabazite pores and that these hydronium ions do not diffuse out of the zeolite pores. Hydrophilic fumed SiO_2_ and γ-Al_2_O_3_ did not catalyse dehydration at measurable rates (Supplementary Table 2).

**Figure 2:**
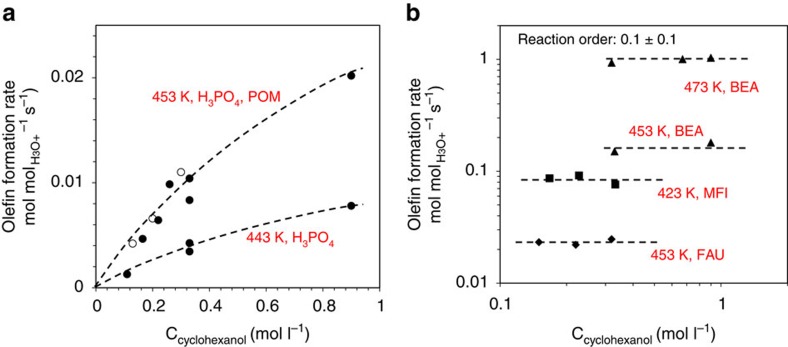
Turnover frequencies of aqueous-phase cyclohexanol dehydration on different acid catalysts as a function of cyclohexanol concentration. (**a**) Soluble acids (closed circles: H_3_PO_4_, open circles: heteropoly acids); (**b**) MFI, BEA and FAU zeolites. Rates were determined at conversions <10%. Dashed lines serve to guide the eye.

In aqueous acidic solutions, the reaction order in cyclohexanol transitioned from first order to fractional order with increasing concentration (0.1–1.2 M; Fig. 2a). In contrast, with medium- and large-pore zeolites, turnover rates did not change with cyclohexanol concentration (Fig. 2b). Note that the first-order kinetics observed for CHA (Supplementary Fig. 2) also contrasted the zero-order kinetics observed for other three zeolites (MFI, BEA and FAU).

Reactions in aqueous acidic media did not yield detectable levels of dicyclohexyl ether or cyclohexylcyclohexenes under the conditions studied. The absence of bimolecular reactions on these acids, even up to the highest residence time when the alcohol−olefin equilibrium was established (Supplementary Fig. 3), is attributed to the low reactant concentrations at the hydronium ion. In contrast, ether formation was readily observed on MFI, BEA and FAU zeolites, with larger pores affording higher ether selectivities (Supplementary Fig. 4).

### Adsorbed states of cyclohexanol and water in zeolite pores

The uptake of cyclohexanol on MFI, BEA and FAU zeolites conformed to the Langmuir-type adsorption isotherm (see Supplementary Fig. 5 for an example). Adsorption equilibrium constants (*K*_ads_°) and saturation uptakes (*q*_max_) were derived using a single-site Langmuir adsorption model to fit the measured isotherms (Table 1). Adsorption enthalpies (Δ*H*_ads_°) were derived from the Van't Hoff plot of *K*_ads_°. The values are in excellent agreement with those determined from aqueous-phase calorimetry (Supplementary Fig. 6). Notably, the heat of adsorption decreased with increasing pore dimensions. The entropy of adsorption (Δ*S*_ads_°) showed the opposite trend, growing more positive with increasing pore size. The more negative enthalpy and entropy of adsorption observed for MFI than larger-pore zeolites are mainly a consequence of more favourable dispersive interactions between the MFI framework and the aliphatic portion of the alcohol. Remarkably, an endothermic, entropy-driven process was observed for the adsorption on zeolite FAU (Table 1). The positive enthalpy and entropy change for adsorption on FAU suggests that cyclohexanol is less well solvated in FAU zeolite than in aqueous solution (see Supplementary Note 2 for related discussions).

The saturation uptake (*q*_max_) became moderately lower at higher temperatures for all zeolites (Supplementary Table 3), as a result of the thermal expansion of the adsorbed phase in the pores^27^. The uptakes at reaction conditions were estimated by extrapolating *q*_max_ to the reaction temperature based on its temperature dependence (measured in the range of 280–353 K, Supplementary Note 3). The maximum uptake of cyclohexanol in MFI at 433–473 K would correspond to ∼2 molecules, on average, per unit cell. In comparison, BEA contains ∼5 cyclohexanol per unit cell, and FAU contains ∼14 cyclohexanol per unit cell (∼2 cyclohexanol molecules per supercage) at saturation under reaction conditions. Assuming that the remaining micropore volume is filled by adsorbed water, the uptake of water in the pore would be 3.8, 4.0 and 6.2 mmol g^−1^, respectively, for MFI, BEA and FAU at 433–473 K (compared with 3.0, 1.8 and 5.9 mmol g^−1^ at room temperature). These upper-bound estimates correspond to ∼20 water molecules per unit cell for both MFI (Si/Al=45, H^+^/u.c.=2) and BEA (Si/Al=75, H^+^/u.c.=0.8) samples, and ∼70 water molecules per unit cell for FAU (Si/Al=30, H^+^/u.c.=2). At this level of pore hydration, hydronium ions form locally in the zeolite pore paired with the AlO_4_^–^ T-site, as shown by X-ray absorption spectroscopy^16^. More importantly, the ratio of intraporous cyclohexanol and water at typical reaction conditions is at least ∼0.1, 0.25 and 0.2, all being much greater than the ratio of cyclohexanol and water in the solution (∼5.6 × 10^−3^ at 0.32 M).

Adsorption constants at reaction temperature were estimated by extrapolation of measured *K*_ads_° on the basis of its temperature dependence (Δ*H*_ads_°). With the extrapolated *K*_ads_° (Supplementary Fig. 7) and the saturation uptakes described above, the fractional uptake (*Θ*, *q*/*q*_max_) was estimated to be high under reaction conditions (0.6–1.0; Table 1). Since the accessible hydronium ions are lower in concentration (0.13–0.27 mmol g^−1^, determined by *in situ* titration) than the saturation uptake (0.40–1.26 mmol g^−1^ at reaction temperatures), we conclude that these hydronium ions are all associated with cyclohexanol under reaction conditions, in line with the zero-order dependence on cyclohexanol concentration (Fig. 2b).

### On the mechanism of aqueous-phase cyclohexanol dehydration

Kinetic H/D isotope effects (KIEs) were used together with ^16^O–^18^O exchange experiments to address whether hydronium ions in solution and in zeolite pores catalyse the dehydration via a common mechanism. Primary KIEs were observed for olefin formation (Supplementary Table 4). The dependence of rates on substrate concentration did not change with H/D substitution (Supplementary Fig. 8). The representative mass spectrometry (MS) fragmentation patterns for the reaction mixtures are presented in Supplementary Fig. 9. The large KIEs are consistent with either an E2 mechanism or an E1 mechanism with a kinetically relevant C–H bond breaking step.

The results of reactions with unlabelled cyclohexanol and H_2_^18^O as solvent (Table 2) and the fact that hydration of olefin hardly occurred under the applied conditions (Supplementary Notes 1, 2 and 4) allow us to conclude that an E2 pathway alone, with concerted C–O and C–H bond scissions, cannot account for the significant ^18^O incorporation (10–32%) into cyclohexanol. With the S_N_2 path for ^16^O–^18^O exchange between water and secondary/tertiary alcohols also ruled out^28^,^29^,^30^,^31^,^32^, the only possible pathway for this level of ^18^O incorporation would be the recombination between H_2_^18^O and an intermediate that is formed upon the C–O bond cleavage and precedes the C_β_–H bond cleavage transition state (TS). Therefore, we conclude that dehydration of cyclohexanol in aqueous phase proceeds along the E1 path. We surmise that this positively charged carbenium ion intermediate is loosely solvated by intrazeolitic water molecules^33^ that act in the final reaction step as the deprotonating base.

**Figure 3:**
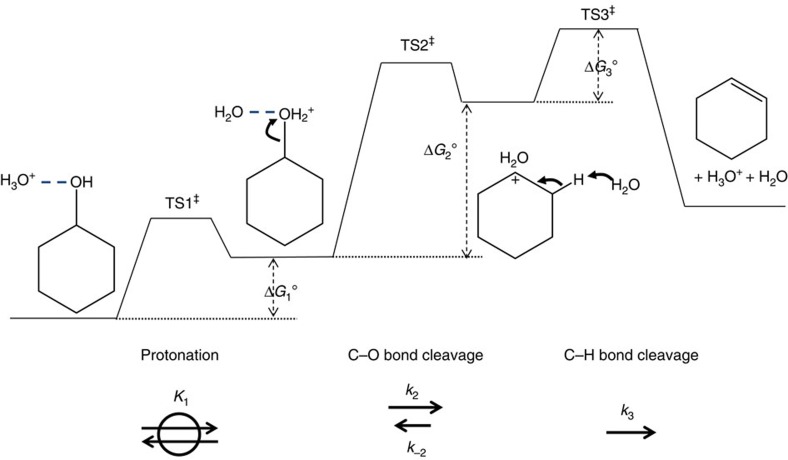
Schematic representation of free energy profile for aqueous-phase cyclohexanol dehydration via an E1 elimination pathway. The reactions are catalysed by hydrated hydronium ions, abbreviated as H_3_O^+^, either in solution or in pores. In aqueous solutions, H_2_O is the most abundant species to deprotonate the carbenium ion. Under our reaction conditions, protonation is quasi-equilibrated, C–O bond cleavage is reversible, and C–H bond cleavage is irreversible. In this work, *k*_-2_ was never much smaller than *k*_3_. For a boundary situation where *k*_3_ is much smaller than *k*_-2_, the measured free energy would be the sum of Δ*G*_1_°, Δ*G*_2_° and Δ*G*_3_°.

Above we concluded that the hydronium ion catalysed dehydration of cyclohexanol occurs via an E1 path with the C_β_–H bond breaking step being kinetically relevant. Figure 3 depicts the free energy diagram for such a mechanism, from which a generalized rate equation is derived (equation (1); derivations in Supplementary Note 5). The rate expression appears complex, as the steady-state assumption has been invoked to replace the concentration term of the carbenium ion intermediate between TS2 and TS3 (Fig. 3).


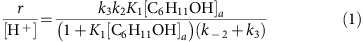


Here, [C_6_H_11_OH]_a_ is the concentration of the association complex formed between cyclohexanol and hydronium ion that depends on the alcohol–hydronium ion association equilibrium constant (*K*_L,a_) and the initial concentration of cyclohexanol. For dilute homogeneous acids ([H_3_O^+^_aq_]=3–4 × 10^−3^ M), *K*_L,a_ was determined to be ∼40 and weakly temperature dependent (433–473 K)^34^. Consequently, at low alcohol concentrations (for example, 0.1 M for H/D isotope experiments), the concentration of the association complex is proportional to the total alcohol concentration. In addition, the protonation equilibrium constant (*K*_1_) for cyclohexanol in water was determined to be the highest (1 M^−1^) at room temperature and then decreased with temperature^35^. At reaction temperatures (for example, 433 K), *K*_1_ (protonation of cyclohexanol) was estimated to be <0.2 M^−1^. Thus, for catalysis by homogeneous acids, equation (1) simplifies to:


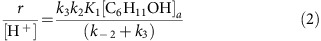


For MFI, BEA and FAU zeolites, we have demonstrated that [C_6_H_11_OH]_a_ inside the zeolite pore does not depend on the alcohol concentration in solution. Since protonation of cyclohexanol by the hydronium ion cluster in zeolites is thermodynamically unfavourable according to density functional theory (DFT) calculations (Supplementary Table 5), equation (2) would also hold true for zeolites. Consequently, the measured KIE on both homogeneous and solid acids is:


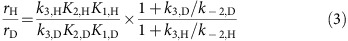


A detailed assessment of the effects of H/D substitution on individual rate and equilibrium constants from statistical thermodynamics (Supplementary Note 6) shows that *k*_3_ (C_β_–H bond cleavage) should be smaller than or comparable to, but cannot be considerably greater than, *k*_-2_ (C_α_–O bond recombination) and that the free energy of TS3 is higher than or similar to that of TS2 (Fig. 3). This is also consistent with our previous analysis of *in situ* nuclear magnetic resonance (NMR) spectroscopic data for this reaction in zeolite HBEA that showed that *k*_−2_ is more than a factor of two greater than *k*_3_ (ref. 23).

We also conclude that the cleavage of a C_β_–H bond is rate determining, the TS of which occurs late along the reaction coordinate and has a free energy similar to or higher than that of the C_α_–O bond cleavage TS (Supplementary Note 6). Remarkably, this finding is in contrast to previous conclusions for gas-phase dehydration of C_2_–C_4_ alcohols on a variety of solid acids^36^,^37^,^38^,^39^, where either an E2 pathway (for Lewis-acidic metal oxides^40^,^41^,^42^) or an E1 pathway with C–O bond cleavage being irreversible and rate determining (for heteropoly acids^36^ and zeolites^37^,^43^) was identified. In particular, we note that for gas-phase alcohol dehydration over solid Brønsted acids, the intermediate state between C–O and C_β_–H bond scissions is typically proposed to be a stable surface alkoxide species rendering the C–O bond cleavage irreversible^44^,^45^. In aqueous-phase dehydration of alcohols, the surface alkoxide is not stable^46^ and is replaced in the reaction pathway by a carbenium ion stabilized by water molecules. We note that the free energy profile illustrated in Fig. 3, purely based on kinetic and isotope measurements, is remarkably consistent with DFT calculations shown for aqueous phase (mimicked by hydronium ion and 20 water molecules inside the pore) cyclohexanol dehydration on zeolite HBEA^34^ and 1-propanol dehydration on zeolite HZSM-5 (ref. 46) via E1-type pathways.

## Discussion

Hydrated hydronium ions are the most acidic species in aqueous acidic solutions; therefore, the hydronium ion-normalized rate of an acid-catalysed reaction, such as the alcohol dehydration studied here, is independent of the intrinsic strength of the dissolved acid. This holds true not only for cyclohexanol (Fig. 1), but also for hydronium ion-catalysed dehydration of substituted cyclohexanols and even more complex molecules such as xylose^13^ (see Supplementary Note 7, where thoughts about rational design of acid catalysts for dehydration of alcohols in aqueous phase are also provided). Under such conditions, the association between hydrated hydronium ions and the reacting substrate is weak, and thus the reaction shows a nearly first-order kinetic behaviour (Fig. 2). In the present case, only 15–19% of the hydronium ions were on time average associated with cyclohexanol in a solution containing 0.02 M H_3_PO_4_ and 0.32 M cyclohexanol at reaction conditions^34^. Full alcohol–hydronium ion association in aqueous solutions would require alcohol concentrations of more than 5 M cyclohexanol. In contrast, this association was nearly complete in a pore of ∼0.7 nm in zeolite BEA, with the ratio of [cyclohexanol] to [water] being more than 20 times larger in the pore than in solution. Thus, molecularly sized confines create a unique nanoenvironment favouring specific alcohol–hydronium ion interactions. The specific substrate-active centre interaction in this nanoenvironment depends markedly on the local geometry and the ordering of substrates and solvents in this environment. The process is enthalpically favoured with zeolites MFI and BEA, while it is entropically favoured with zeolite FAU (Table 1).

The enthalpies, entropies and free energies of activation (at 443 K), derived from kinetic measurements and the transition state theory formalism, are compiled in Table 3. In all cases, the magnitude of the entropy gain points to significant product-like character in the TS, interpreted as stabilization of the transition state by the environment. The variations in the entropy of activation, however, should not be simplistically interpreted as an indication of an earlier or later TS, as it was shown that there is significant C_β_–H bond breaking (that is, a late TS) in reaching the kinetically relevant TS (TS3 in Fig. 3, C_α_–O already broken) for all aqueous-phase dehydration catalyses. The differences in Δ*G*°^‡^ (Table 3) indicate that the catalytic activity of hydronium ion is intrinsically lower in the unconstrained aqueous solution than in MFI and BEA zeolites (but not FAU). The intrinsic activity of the intraporous hydronium ions is the lowest for FAU, as indicated by the largest Δ*G*°^‡^. Therefore, the entropy-induced nearly complete association of hydronium ion and alcohol substrate must be solely responsible for the higher TOF on FAU than aqueous acids (Fig. 1).

For zeolite-catalysed dehydration, both Δ*H*°^‡^ and Δ*S*°^‡^ increased with increasing pore size, most prominently from MFI to BEA. This can be qualitatively attributed to the increased van der Waals contacts for the larger-sized TS (loosely bound carbenium ion and water) relative to the adsorbed alcohol with the internal walls of smaller pores. However, it is important to mention that the observed activation enthalpies and entropies may include contributions from changes in the organization of the reactant molecule itself and water molecules before and after reaction^47^ that likely depend on the (liquid or intraporous) environments. Also note that while the extent of additional solvation by intraporous water of intermediates and TS may change with the concentration of hydrophilic entities in the pores, the relatively minor variations in zero-order rate constants on MFI and BEA samples^3^ with different Al concentrations appear to indicate that such intraporous water solvation occurs to similar extents for H-bonded cyclohexanol and the kinetically relevant TS within a given zeolite framework structure.

In conclusion, the catalytic activity of hydronium ion is drastically altered by the constraints and the local organization of solvents and substrate molecules induced by its environment. The compensation between enthalpy and entropy induced by the nanoscopic environment results in a ranking of free energy of activation that perfectly explains the measured activities and allows predictions of activities for other Brønsted acids and alcohol substrates. These findings show that a molecular environment can be tailored to dramatically enhance reaction pathways for acid catalysis in water, opening new opportunities for designing catalysts for selective conversions, utilizing confinement principles in analogy to enzymatic systems.

## Methods

### Catalysts

Commercial zeolites were obtained in the H-form, activated once (1 K min^−1^ to 723 K in a 100 ml min^−1^ flow of dry air for 6 h) and stored in closed containers kept away from volatile organics. Specifically, HZSM-5 (Si/Al=45) and HBEA (Si/Al=75) were provided by Clariant AG, while HFAU (CBV760; Si/Al_total_=30, Si/Al_F_=60) was obtained from Zeolyst International. CHA was synthesized according to the reported procedure^48^. SiO_2_ (Aerosil-90), amorphous silica alumina (ASA, catalyst support grade 135), phosphotungstic acid (H_3_PW) and tungstosilicic acid (H_4_SiW) were purchased in hydrate form from Sigma–Aldrich. Commercial γ-Al_2_O_3_ and boehmite products from Sasol (Condea) were also tested for the title reaction. More details about the chemicals and catalyst characterization protocols are provided in the Supplementary Methods.

### Liquid-phase adsorption and calorimetry

Adsorption isotherms were obtained by immersing 20–100 mg of zeolite in a cyclohexanol solution at a given concentration for 24 h. The liquid was separated from the zeolite by filtration and the residual concentration of cyclohexanol in the solution was determined by liquid-state NMR using the internal standard (1,3,5-trioxane). The uptake was determined by the change in the bulk concentration, volume of the solution and mass of the solid sample. Heat of cyclohexanol adsorption from aqueous solutions into MFI, BEA and FAU zeolites was determined by aqueous-phase calorimetry using a Setaram Calvet C80 calorimeter with reversal mixing cells. The lower compartment was loaded with 0.03 g zeolite dispersed in 0.8 ml water, while the upper compartment was loaded with 0.2 ml of the cyclohexanol solution. The reference cell was loaded with liquids with identical compositions but without zeolite.

### Kinetic measurements

Aqueous-phase dehydration reactions were performed in a 300 ml Hastelloy Parr reactor, a gradientless batch reactor. Preliminary tests showed that the zeolite samples (protected from contaminants) could be used without reactivation procedures. For reactions catalysed by solid acids, the external diffusion limitation (mass transport of the dissolved reactants from the liquid bulk to the outer surface of the catalyst particles) was precluded in preliminary experiments varying the stirring speed (400–700 r.p.m.) and catalyst loading (50–300 mg).

In a typical experiment, 80 ml 0.33 M aqueous cyclohexanol solution and a specific amount of catalyst (for example, 100–200 mg for zeolites) were loaded in the reactor. The closed reactor was then pressurized with 40 bar H_2_ or 6 bar N_2_ (for heteropoly acids due to their reducibility at elevated temperatures) at room temperature and heated up while being stirred vigorously (∼700 r.p.m.). The reaction time was based on the point when the set temperature was reached (∼10–15 min). At the end of a reaction, the reactor was cooled with an ice/water mixture to at least 277 K. The reaction mixture of cyclohexene (oil phase) and cyclohexanol-containing aqueous phase was extracted using dichloromethane (20 ml DCM per extraction, 5 times). The combined DCM phase was dried over Na_2_SO_4_ and an aliquot of the solution was analysed on an Agilent 7890A gas chromatograph (GC) equipped with a HP-5MS 25 m × 0.25 μm (i.d.) column, coupled with Agilent 5975C MS. For quantification, 1,3-dimethoxybenzene was used as the standard. The carbon balance was typically at 85–90%.

For titration experiments, different quantities of aqueous solutions (0.03 M) of pyridine or 2,6-lutidine were added to the starting reaction mixture, and then rates were determined, using the protocol described above, on these pretitrated samples. Pyridine and 2,6-lutidine adsorb much more strongly than cyclohexanol on acid sites (differences in heat of adsorption >20 kJ mol^−1^ according to aqueous-phase calorimetric measurements). Consequently, the uptake of base titrants was always complete, as verified by the analysis of the post-extraction DCM phase by GC–MS (splitless injection).

### H/D kinetic isotope effects and ^18^O-tracer experiments

Rates of dehydration were measured in the Parr reactor for perdeuterated cyclohexanol (0.10–0.11 M, 80 ml solution), using operation protocols described above. Experiments using ^18^O-labelled water and nonlabelled cyclohexanol (0.3 M) were carried out in a ∼2 ml stirred batch reactor constructed from a stainless steel ‘tee' (HiP), while ensuring similar solution-to-headspace ratios (0.3–0.4) as in the Parr reactor. The recovered post-reaction mixture was extracted with DCM (0.5 ml per extraction, 4 times), dried over Na_2_SO_4_ and analysed with GC–MS. For the analysis of ^18^O content in recovered alcohol, it is useful to consider that the dominant MS fragment ion of unlabelled cyclohexanol is *m/z* 57, the [C_3_H_5_O]^+^ ion, while the *m/z* 59 is present with 1% of the ion intensity of *m/z* 57. Thus, the intensity ratio between the two fragment ions can be used to quantify the extent of ^18^O incorporation into cyclohexanol that would lead to a growth of the *m/z* 59 peak.

### Data availability

All data are available within the article (and its Supplementary Information files) and from the authors on reasonable request.

## Additional information

**How to cite this article:** Shi, H. *et al*. Tailoring nanoscopic confines to maximize catalytic activity of hydronium ions. *Nat. Commun.* **8,** 15442 doi: 10.1038/ncomms15442 (2017).

**Publisher's note**: Springer Nature remains neutral with regard to jurisdictional claims in published maps and institutional affiliations.

## Supplementary Material

Supplementary Information Supplementary figures, supplementary tables, supplementary notes, supplementary methods and supplementary references.

## Figures and Tables

**Table 1:** Adsorption parameters for cyclohexanol uptake from aqueous solutions into three zeolites (denoted with the respective Si/Al ratio).*

**Zeolite**	***K***_**ads**_**°**†	***q***_**max**_‡ **(mmol** **g**^−**1**^**)**	**Δ*****H***_**ads**_**° (kJ** **mol**^−**1**^**)**	**Δ*****S***_**ads**_**° (J** **mol**^**−1**^ **K**^**−1**^**)**	***Θ***§
MFI45	1,582 (192)	0.66	–30	–55	0.60–0.69
BEA75	580 (140)	1.60	–22	–25	0.77–0.81
FAU30	51 (67)	1.45	+4	+47	0.96–0.97

^*^Adsorption constants were derived from the slope of the linearized Langmuir isotherm, standard molar enthalpy changes of cyclohexanol adsorption were determined by microcalorimetry (<60% of the saturation uptake) and variable-temperature isotherm measurements and standard molar entropy changes were obtained from Δ*G*_ads_°=−*RT* ln*K*_ads_°=Δ*H*_ads_°−*T*Δ*S*_ads_° that relates all thermodynamic quantities. Standard states for aqueous and adsorbed molecules are 1 mol l^−1^ and intrazeolitic void occupancy=1, respectively.

^†^Adsorption constants at 280 K (outside the brackets) and 333 K (in the brackets).

^‡^Saturation uptake at 298 K.

^§^Predicted fractional uptake (*q*/*q*_max_) at 423–443 K, 0.33 M aqueous solution of cyclohexanol.

**Table 2:** The ^18^O exchange during aqueous-phase cyclohexanol dehydration.*

**Catalyst**	^**18**^**O in the recovered alcohol (%)**†	**Conversion (%)**
MFI, 433 K	32±2	22
BEA, 453 K	10±1	19
H_4_SiW, 453 K	16±1	15

^*^Extent of ^18^O exchange from H_2_^18^O (97% isotopic purity) into unlabelled cyclohexanol and its conversion during dehydration (cyclohexanol concentration: 0.30 M in H_2_^18^O).

^†^Determined from the intensity ratio between fragment ions *m/z* 57 and 59 in the MS fragmentation patterns (Supplementary Fig. 10) and error bars reflect the uncertainties in this ratio.

**Table 3:** Intrinsic activation parameters for aqueous-phase dehydration of cyclohexanol.*

**Catalyst**	**Δ*****H*****°**^**‡**^ **(kJ** **mol**^**−1**^**)**	**Δ*****S*****°**^**‡**^ **(J** **mol**^**−1**^** K**^**−**^**)**	**Δ*****G*****°**^**‡**^_**443K**_ **(kJ** **mol**^**−1**^**)**
MFI	140±5	62±10	112±1
BEA	159±4	87±9	120±1
FAU	166±4	88±10	127±1
Soluble acids	154±4	68±8	124±1

^*^Standard enthalpies, entropies and free energies of activation (at 443 K) on zeolites and homogeneous acids, derived from kinetic measurements and the transition state theory formalism. The error bars for Δ*H*°^‡^ and Δ*S*°^‡^ represent the 1−*σ* s.d.'s, while the error bar for Δ*G*°^‡^ represents the maximum error rounded up to the nearest integer (error analysis protocol detailed in Supplementary Methods).
