# Supplementary material for: Tailoring nanoscopic confines to maximize catalytic activity of hydronium ions
Source: Nat Commun. 2017 May 25;8:15442. doi: 10.1038/ncomms15442 (PMC5458516; doi:10.1038/ncomms15442)
Supplement: Supplementary Information — Supplementary figures, supplementary tables, supplementary notes, supplementary methods and supplementary references. [file ncomms15442-s1.pdf]

Supplementary Table 1 | Textural properties and Brønsted/Lewis acid site concentrations of various solid acids and recovered samples after reaction.

Catalysts	Si/Al ratio	N_2 physisorption			Acidity (μmol/g)[a]
		BET surface area (m^2/g)	V_{micro} (cm^3/g)	V_{meso} (cm^3/g)	
H-BEA	75	597	0.18	0.29	125 ± 10 (B167, L36[b])
Spent H-BEA[c]	n.m.[d]	609	0.16	0.34	110 ± 5
H-MFI-1[e]	45	416	0.12	0.09	260 ± 15 (B309, L25[b])
Spent H-MFI[f]	n.m.[d]	409	0.12	0.09	270 ± 6
H-MFI-2[g]	40	380	0.15	0.06	350 ± 12
H-USY	29	750	0.26	0.15	170 ± 9 (B140, L49[b,h])
Spent H-USY[i]	n.m.[d]	732	0.24	0.18	n.m.[d]
H-CHA	35	805	0.31	0.02	n.m.[d]
H-MFI-AHFS[j]	26	427	0.16	0.12	n.m. (620, 62[b])

[a] Acidity obtained from in situ titration experiments using pyridine and 2,6-lutidine; see Experimental for details.
[b] Values in parentheses (B,L) represent concentrations of Brønsted (1540 cm^{-1}, molar integral extinction coefficient of 0.73 cm μmol^{-1}) and Lewis acid sites (1450 cm^{-1}, molar integral extinction coefficient of 0.96 cm μmol^{-1}) determined from gas-phase IR measurements using pyridine at 423 K.
[c] Recovered sample after 1 h reaction at 453 K.
[d] Not measured.
[e] From Clariant; 0.2–0.5 μm particle size.
[f] Recovered sample after 1 h reaction at 423 K.
[g] From Zeolyst; ~ 1 μm particle size.
[h] From ref.[1].
[i] Recovered sample after 1 h reaction at 453 K.
[j] A sample obtained from $(NH_4)_2SiF_6$ treatment of an NH_4-MFI sample (Zeolyst International, CBV3024E, Si/Al = 15) and subsequently activated (calcination at 823 K for 5 h in 100 mL min^{-1} synthetic air with a heating rate of 10 K min^{-1}).

Supplementary Table 2 | Mass specific rates and turnover rates of cyclohexanol dehydration on Al_2O_3, SiO_2, chabazite zeolite (CHA) and without catalyst in water at 473 K.

Catalyst	Mass specific rates (mol g^{-1} s^{-1})	Turnover rate (mol mol_{H+} s^{-1})
CHA	4.5×10^{-7}	-
Al_2O_3	No activity[a]	No activity[a]
SiO_2	No activity[a]	No activity[a]
H_3PO_4	–[b]	5.5×10^{-2}
No catalyst (pure water)	No activity[a]	No activity[a]

[a] No detectable conversion using 1 g of catalyst, 4 h reaction at 473 K. [b] Volumetric rate 1.5×10^{-4} M s^{-1}.

Supplementary Table 3 | Saturation uptake measured from adsorption isotherms of cyclohexanol from aqueous solutions to zeolites.

Adsorption temperature (K)	Saturation uptake (mmol g^{-1})		
	HMFI45	HBEA75	HFAU30
280	0.68	1.75	1.47
298	0.66	1.60	1.45
313	0.63	n.d.[a]	1.44
323	n.d.[a]	1.50	n.d.[a]
333	n.d.[a]	1.52	1.42
353	0.60	1.31	1.40

[a] Not determined

Supplementary Table 4 | H/D isotope effects measured over different acid catalysts.[a] Part of the results (BEA and H_3PO_4) were recently reported.[2]

Reactant	TOF (10^{-3} s^{-1})		
	MFI[b]	BEA[c]	H_3PO_4[d]
$C_6H_{11}OH$	83 ± 5	55 ± 3	3.5 ± 0.2
$C_6D_{11}OH$	24 ± 2	19 ± 1	1.2 ± 0.1
KIE	3.5 ± 0.5	2.9 ± 0.3	3.0 ± 0.4

[a] Reactant conversions were kept at 5–20% and dicyclohexyl ether selectivities at 0–2%; cyclohexanol and perdeuterated cyclohexanol (forming $C_6D_{11}OH$ upon exchange with H_2O) were dissolved in unlabeled water (~ 0.1 M); 98 atom% isotopic purity for $C_6D_{11}OD$.; 98 atom% isotopic purity for $C_6D_{11}OD$. [b] At 423 K. [c] At 443 K. [d] At 453 K.

Supplementary Table 5 | DFT estimates of protonation constant of adsorbed cyclohexanol in periodic MFI (Si/Al = 47) and BEA (Si/Al = 63) structures. The models for DFT calculations have been described in detail in our recent works.[2,3] $C_6H_{11}OH + H^+(H_2O)_8 \leftrightarrow C_6H_{11}OH_2^+ + (H_2O)_8$

Zeolite	ΔG°	$K_{prot}°$
MFI	+22	2.5×10^{-3}
BEA	+8	0.12

Supplementary Table 6 | Measured turnover rates (at conversions < 10%; normalized to the concentration of Brønsted acid sites) of cyclohexanol dehydration (concentration: 0.33 M at room temperature) in the presence or absence of 10%Pd/Al_2O_3 that catalyze hydrogenation of the olefin product.

Acid catalyst and temperature	Turnover rate (10^{-2} s^{-1})	
	Without Pd	With Pd
H-Beta, 443 K	6.1 ± 0.3	6.6 ± 0.2
H-Beta, 453 K	15.2 ± 1.0	17.0 ± 1.3
H-ZSM-5, 423 K	7.5 ± 0.5	8.1 ± 0.4
$H_3PW_{12}O_{40}$, 443 K	0.36 ± 0.02	0.40 ± 0.03

Supplementary Table 7 | Single ion peak intensity (normalized to the intensity of the m/z 92 ion fragment) in the MS patterns (70 eV) for the recovered reaction mixture (after extraction into dichloromethane) after catalyzed reactions on MFI, BEA and H_3PO_4.

m/z	No reaction	MFI (conv. 9%)	BEA (conv. 11%)	H_3PO_4 (conv. 18%)
90	0.10	0.16	0.11	0.11
91	0.36	0.45	0.38	0.41
92	1.0	1.0	1.0	1.0

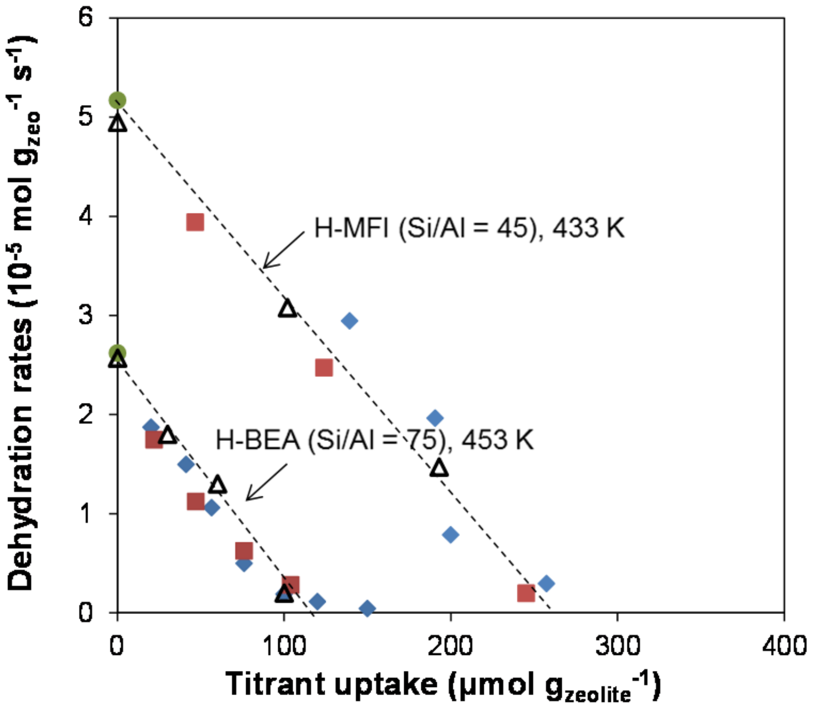

Supplementary Figure 1 | Mass specific rates for cyclohexanol dehydration on MFI and BEA zeolites as a function of the titrant uptake. Green circles are rates on fresh catalysts, red squares correspond to rates after titration using 2,6-lutidine, blue diamonds correspond to rates after titration using pyridine, while black triangles correspond to pyridine titration experiments on recycled catalysts. Dashed lines are shown as guide to the eyes.

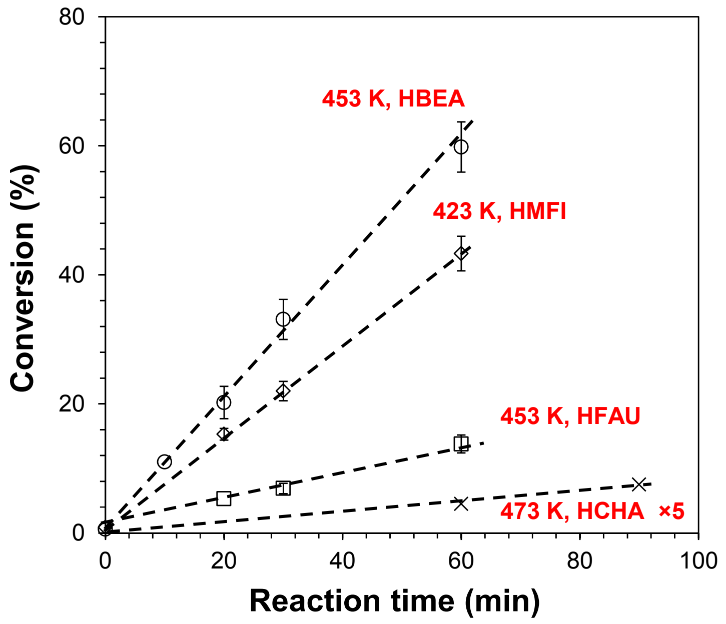

Supplementary Figure 2 | Cyclohexanol conversion on various solid acid catalysts as a function of reaction time. The mass of catalyst used: 0.2 g (HBEA), 0.15 g (HMFI), 0.2 g (HFAU) and 0.1 g (HCHA). For the sake of clarity, the conversion value (y-axis value) on HCHA was increased by a factor of 5. Dashed lines are shown as guides to eyes. This plot shows that all solid acids are functionally intact within the studied time scale, as otherwise, if structural destruction had occurred, conversion would not have increased linearly with reaction time. The error bars reflect the experimental uncertainties based on multiple measurements. Rates are compiled in Supplementary Table 2.

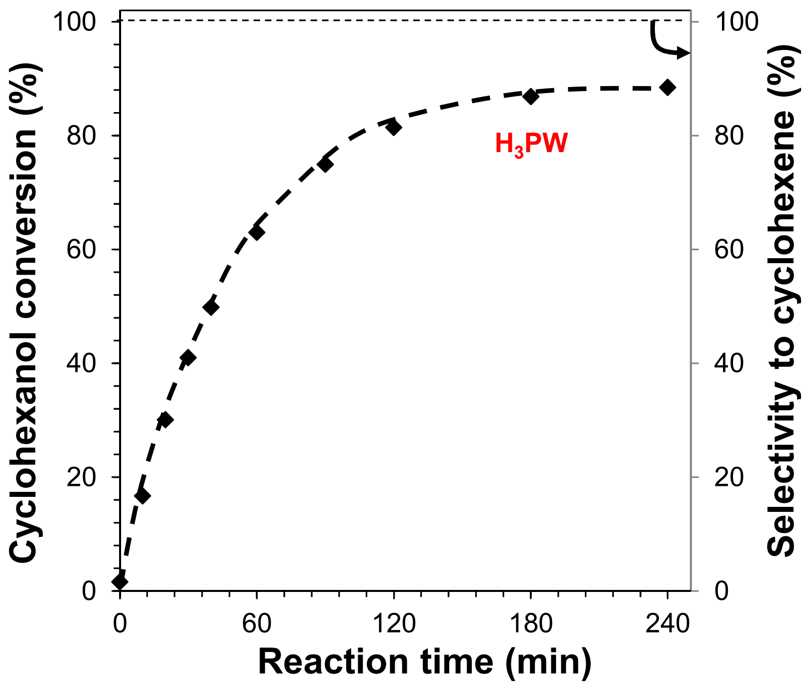

Supplementary Figure 3 | Conversion of cyclohexanol (0.33 M aqueous solution) and the selectivity to cyclohexene (staying 100%) towards the equilibrium of dehydration at 473 K, catalyzed by $H_3PW_{12}O_{40}$ (6×10^{-4} M). All concentrations are reported on the basis of the solution volume at room temperature, not correcting for density change of the liquid mixture at 473 K.

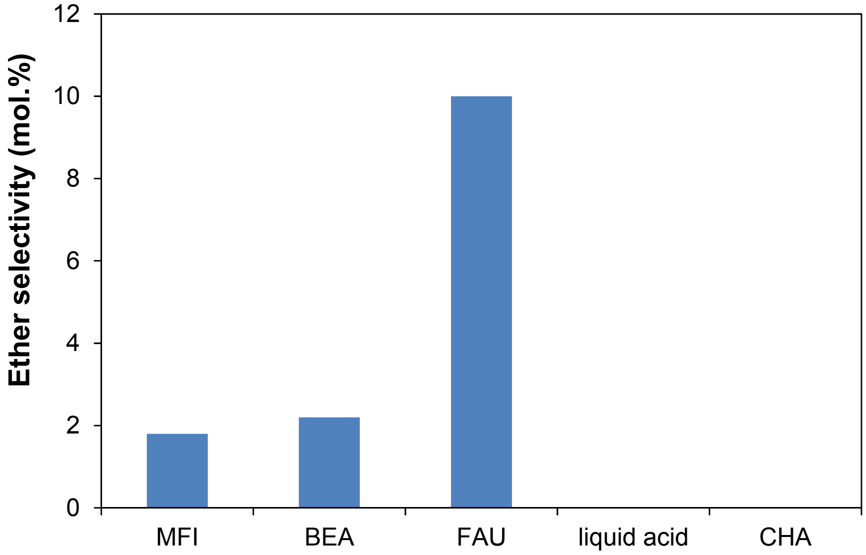

Supplementary Figure 4 | Selectivities to dicyclohexyl ether over various acid catalysts at short residence times (< 1 h, similar trends for all temperatures). "Liquid acids" include $HClO_4$, H_3PO_4, $H_3PW_{12}O_{40}$ and $H_4SiW_{12}O_{40}$.

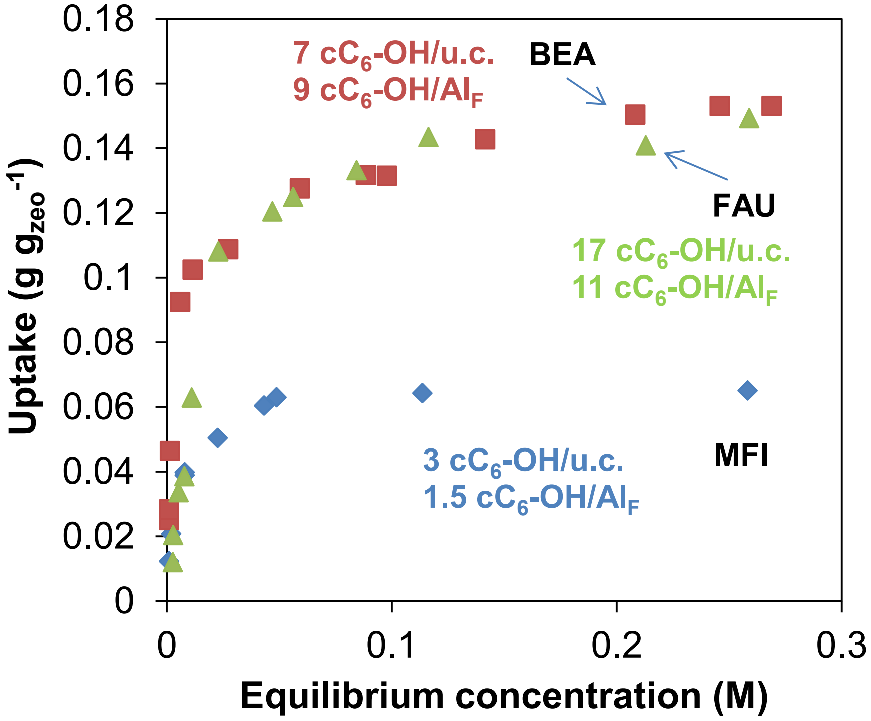

Supplementary Figure 5 | Adsorption isotherms of cyclohexanol from aqueous solutions onto/into MFI (Si/Al = 45), BEA (Si/Al = 75) and FAU (Si/Al = 30) zeolites at 298 K.

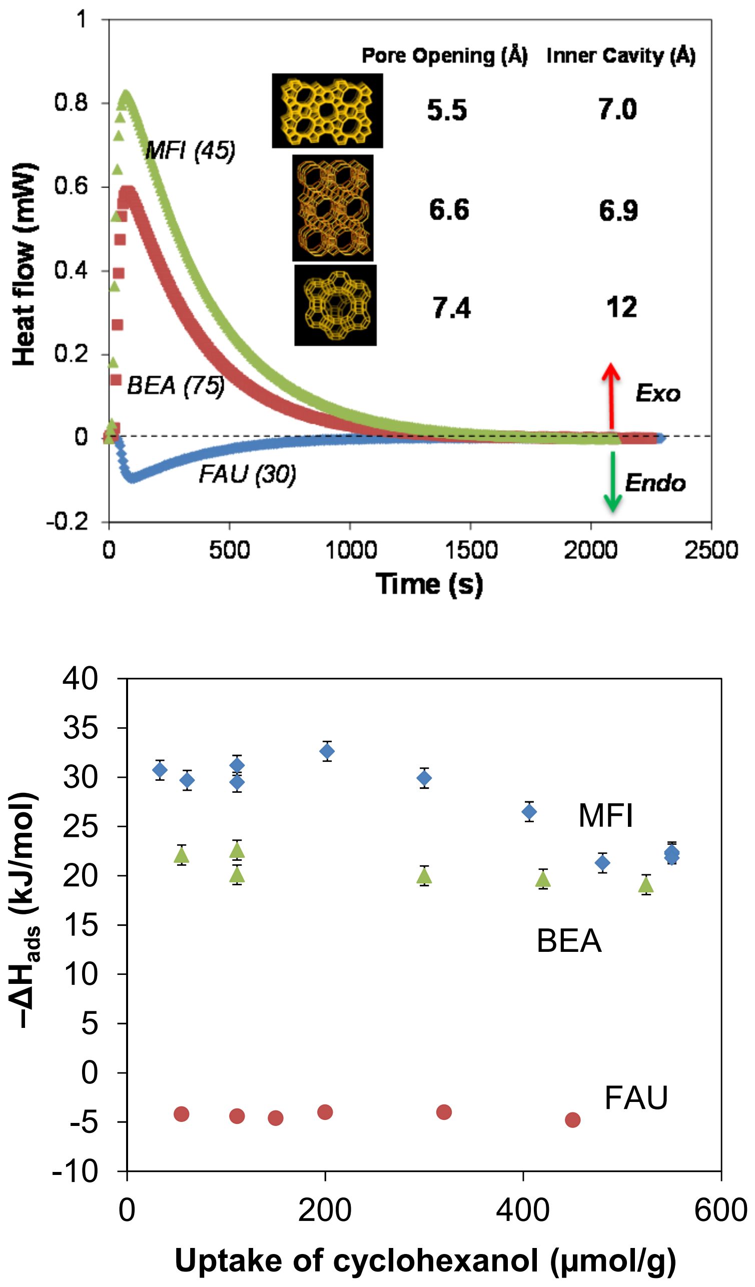

Supplementary Figure 6 | Calorimetric measurements of cyclohexanol adsorption from aqueous solutions to zeolites. Top panel: Heat evolution as a function of mixing time during calorimetric measurements of cyclohexanol uptake from dilute aqueous solutions onto MFI, BEA and FAU zeolites (the corresponding Si/Al ratios are denoted in the parentheses). The insets show the pore topologies with the sizes of pore opening and the largest inner cavity. Bottom panel: Heat of adsorption as a function of cyclohexanol uptake from aqueous solutions on MFI, BEA and FAU. The error bars reflect uncertainties in the integration of heat signals. All measurements were made at 298 K.

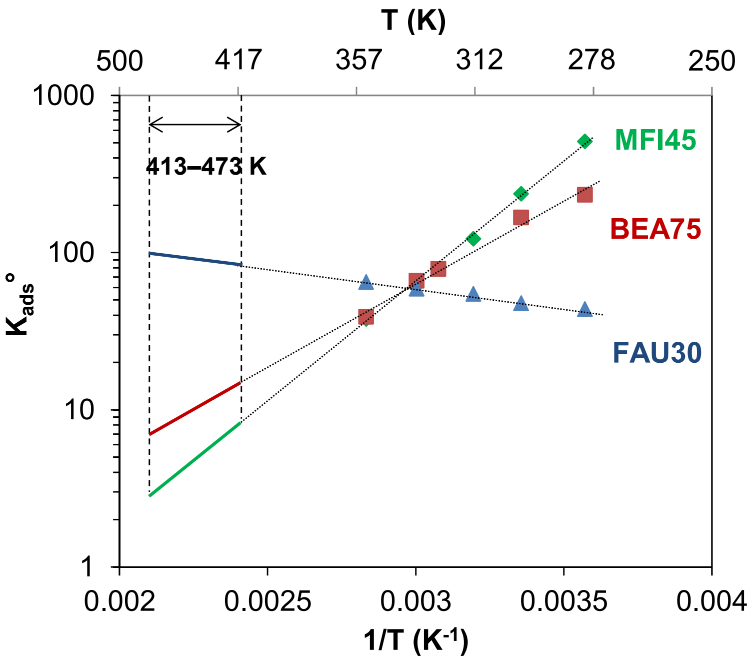

Supplementary Figure 7 | Measured (solid symbols) adsorption equilibrium constant (K_{ads}°), as well as the extrapolated values (solid lines) at reaction temperatures, for cyclohexanol adsorption from aqueous solutions onto zeolites. Si/Al ratios are denoted after the framework type code.

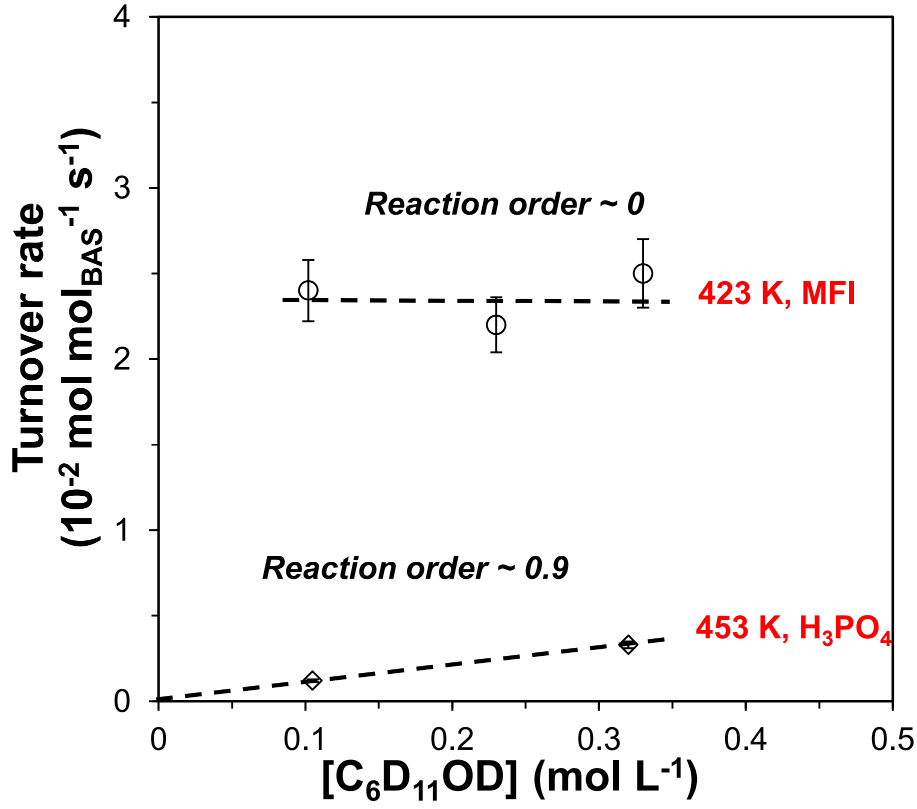

Supplementary Figure 8 | Dependence of dehydration rates on the concentration of perdeuterated cyclohexanol (in the form of $C_6D_{11}OH$ in H_2O) over MFI and H_3PO_4. The error bars in rates reflect the uncertainties based on linear regression fits of the conversion-time plots with 95% confidence level. Dashed lines are shown as guides to eyes.

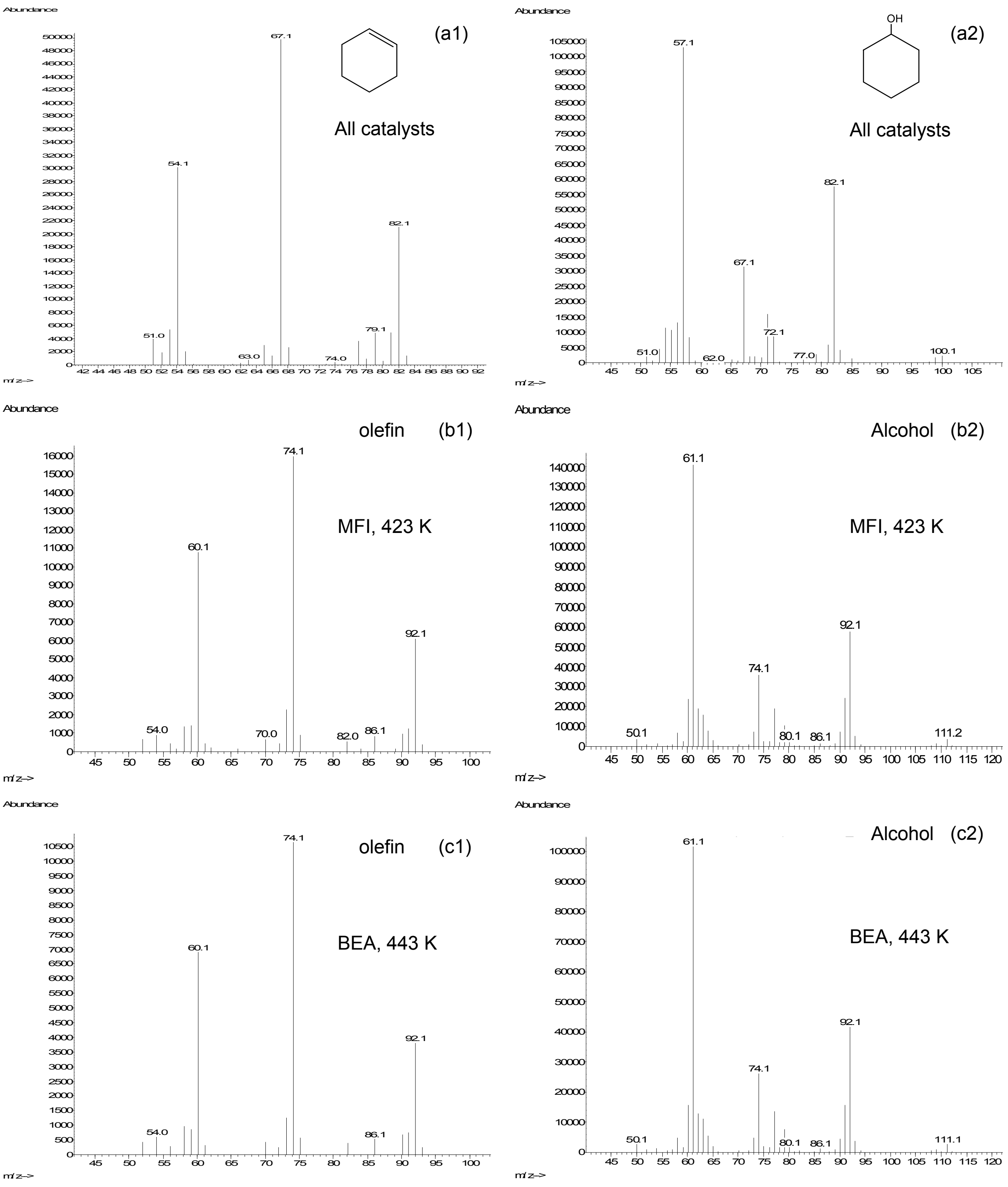
Abundance
(a1)
All catalysts
51.0
54.1
63.0
67.1
74.0
79.1
82.1
m/z-->
Abundance
OH
(a2)
All catalysts
51.0
57.1
62.0
67.1
72.1
77.0
82.1
100.1
m/z-->
Abundance
olefin (b1)
MFI, 423 K
54.0
60.1
70.0
74.1
82.0
86.1
92.1
m/z-->
Abundance
Alcohol (b2)
MFI, 423 K
50.1
61.1
74.1
80.1
86.1
92.1
111.2
m/z-->
Abundance
olefin (c1)
BEA, 443 K
54.0
60.1
74.1
86.1
92.1
m/z-->
Abundance
Alcohol (c2)
BEA, 443 K
50.1
61.1
74.1
80.1
86.1
92.1
111.1
m/z-->

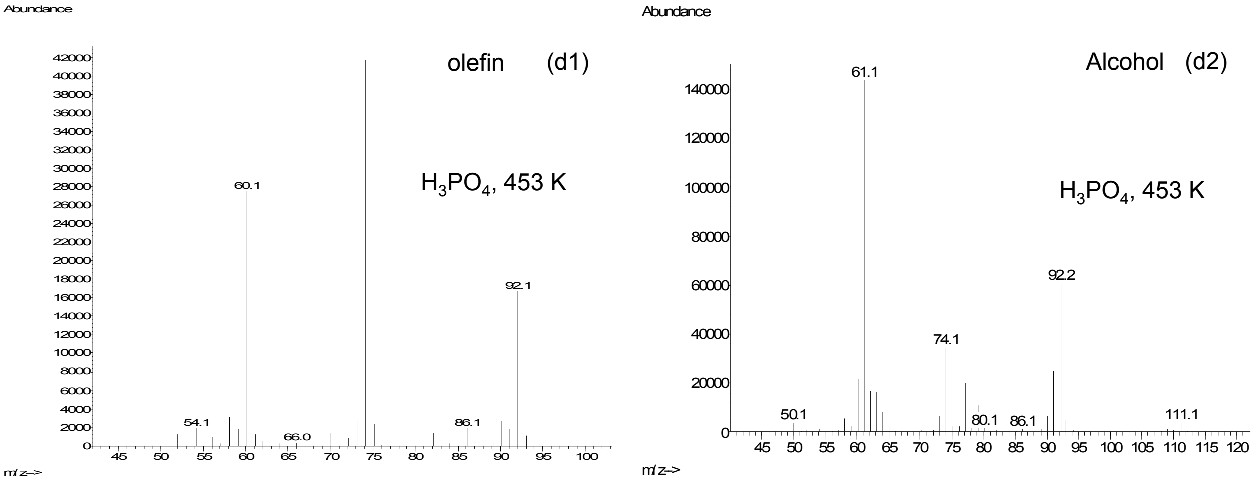

Supplementary Figure 9 | Representative MS fragmentation patterns of the recovered reaction mixture (after extraction with DCM) for aqueous-phase dehydration of cyclohexanol-d_0 (a) and cyclohexanol-d_{12} (b–d) over different acid catalysts. (a1–d1) and (a2–d2) correspond to the olefin product and alcohol reactant, respectively. Catalysts and temperature used are denoted in each figure. Solvent was unlabeled water in all cases.

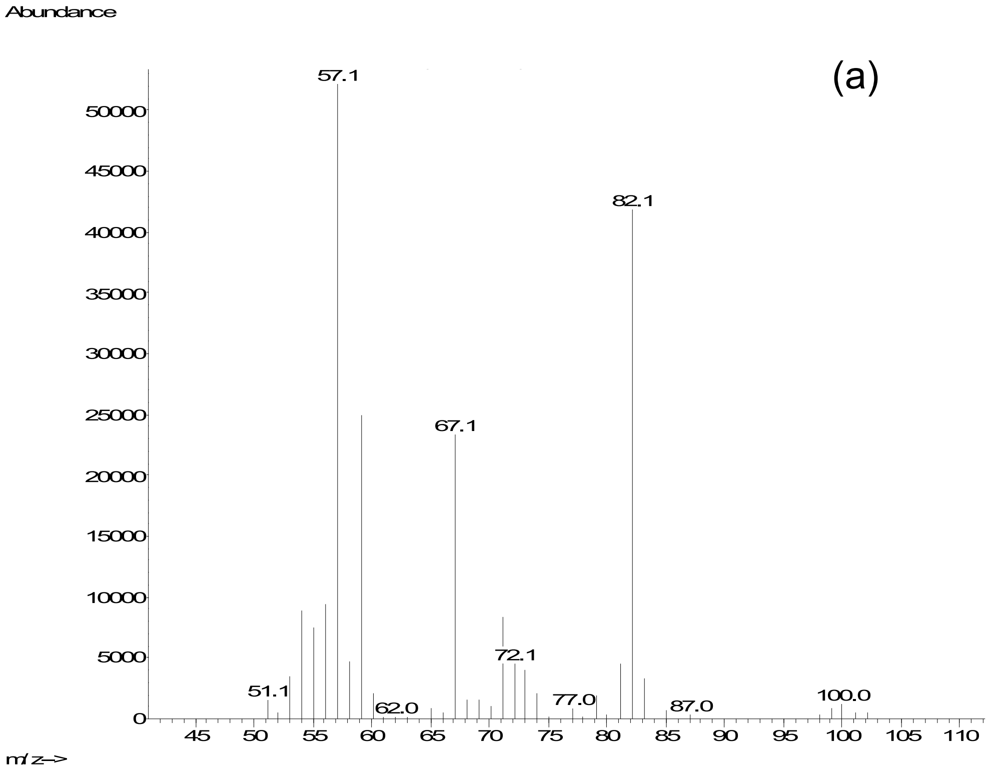
Abundance
(a)
57.1
82.1
67.1
72.1
51.1
62.0
77.0
87.0
100.0
50000
45000
40000
35000
30000
25000
20000
15000
10000
5000
0
45 50 55 60 65 70 75 80 85 90 95 100 105 110
m/z-->

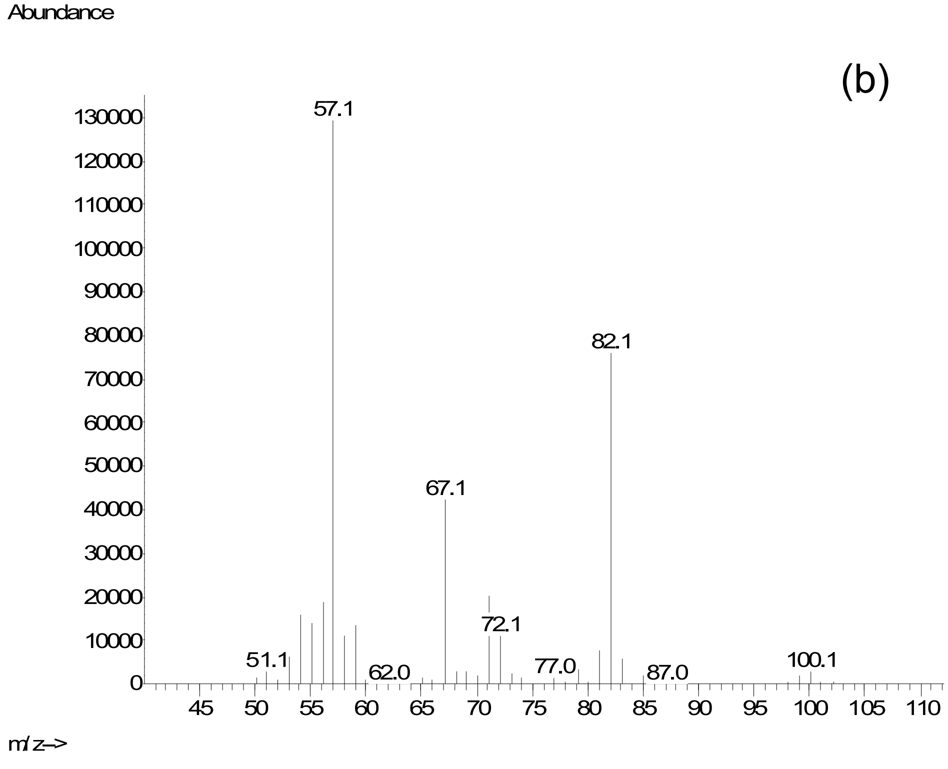
Abundance
(b)
57.1
82.1
67.1
72.1
51.1
62.0
77.0
87.0
100.1
130000
120000
110000
100000
90000
80000
70000
60000
50000
40000
30000
20000
10000
0
45 50 55 60 65 70 75 80 85 90 95 100 105 110
m/z-->

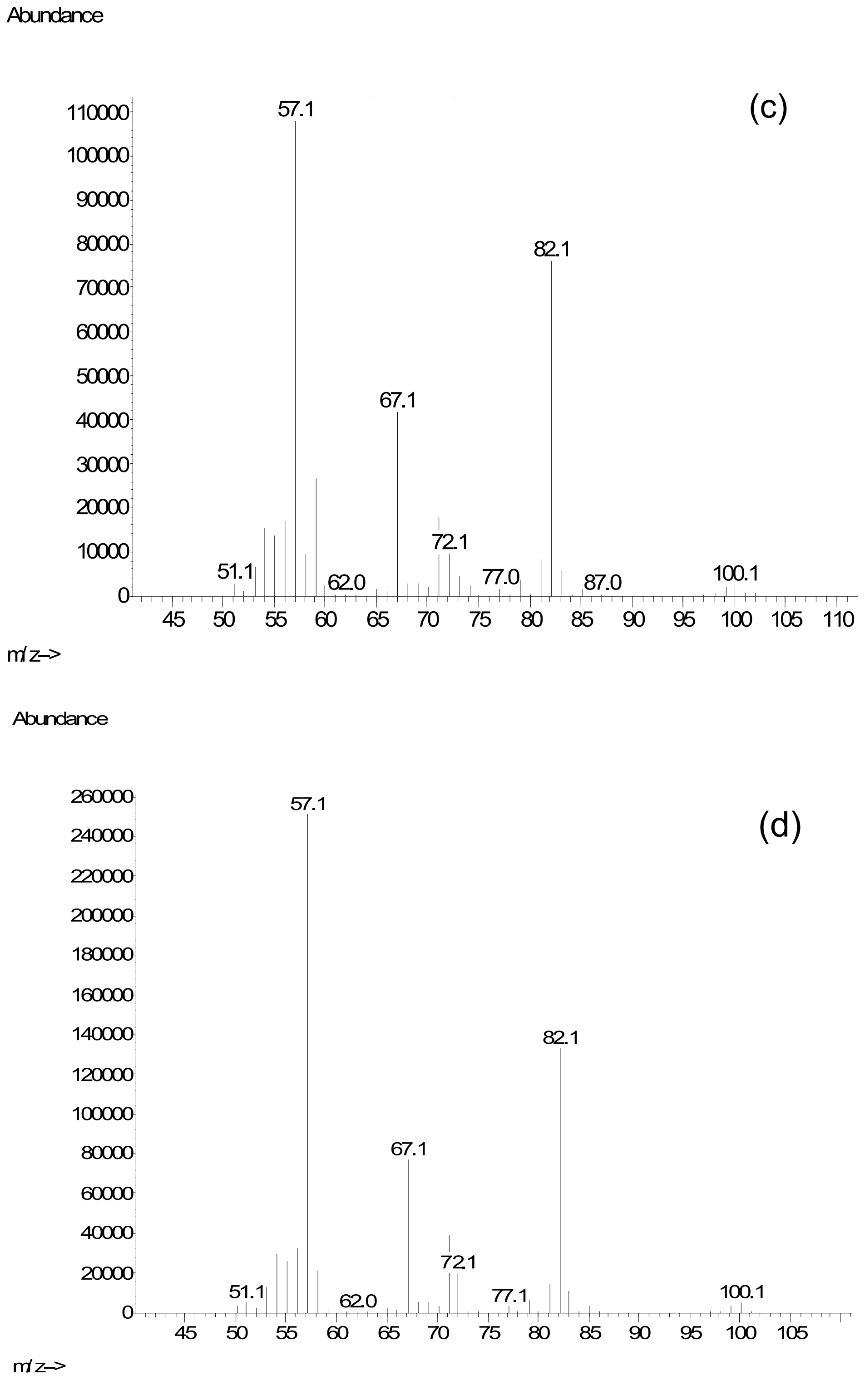

Supplementary Figure 10 | Representative MS fragmentation patterns of recovered cyclohexanol after ^{18}O-exchange reactions catalyzed by (a) MFI; (b) BEA and (c) $H_4SiW_{12}O_{40}$. (d) is for the as-received unlabeled cyclohexanol.

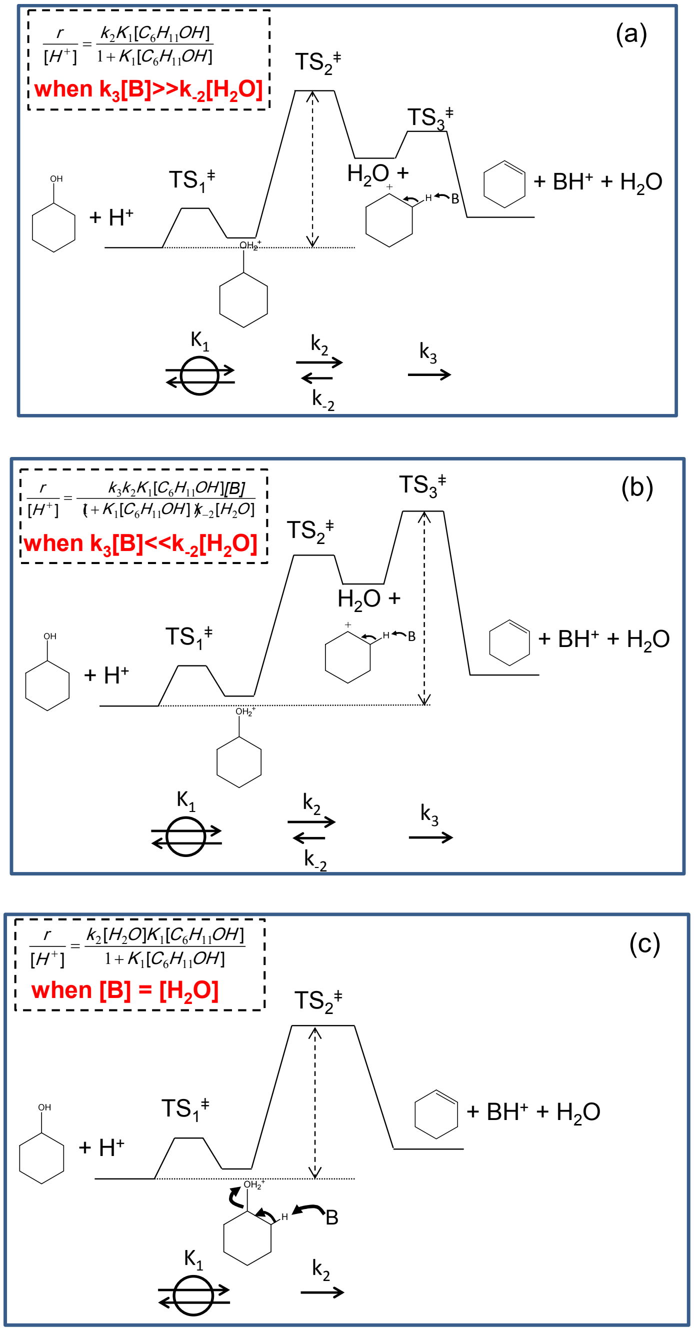

Supplementary Figure 11 | Schematic representations of free energy diagram for cyclohexanol dehydration via classical elimination pathways: (a) and (b) are E1-type paths while (c) is an E2-type path. In aqueous phase, the Brønsted acid site (H^+) is effectively a hydronium ion. [B] is the base that accepts the proton; in an aqueous solution without external bases, H_2O is the most abundant base. Rate expressions are shown for simplified cases, while more general derivations are shown in Supplementary Note 5. The TS that is associated with the rate constant appearing in the rate expression is the kinetically relevant TS, e.g., TS2 in case (a) and TS3 in case (b).

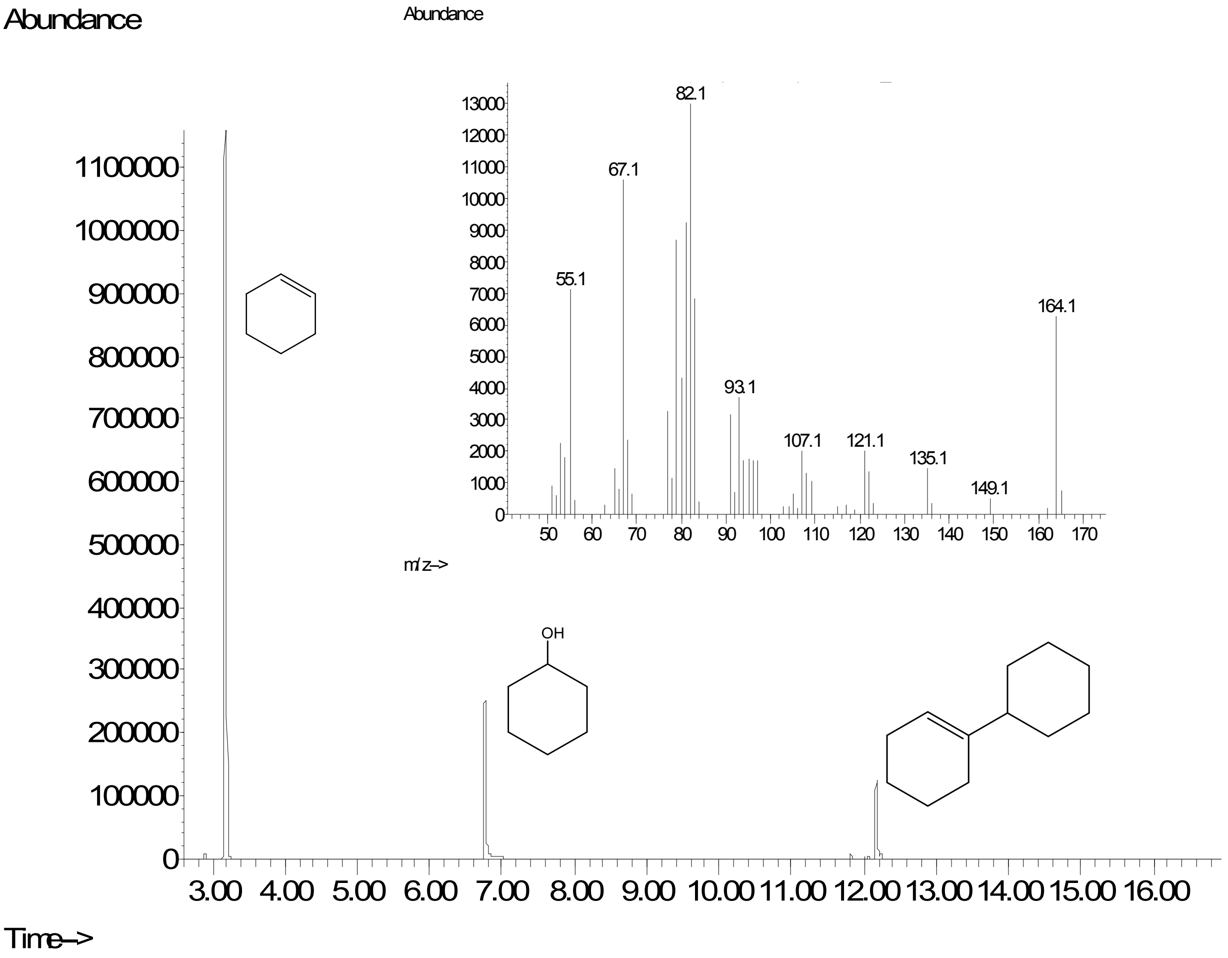

Supplementary Figure 12 | A representative GC profile of the reaction mixture after cyclohexene hydration at 443 K for 1 h (1 g of cyclohexene, 80 ml water and 0.2 g HBEA). The inset shows the MS pattern of 1-cyclohexylcyclohexene.

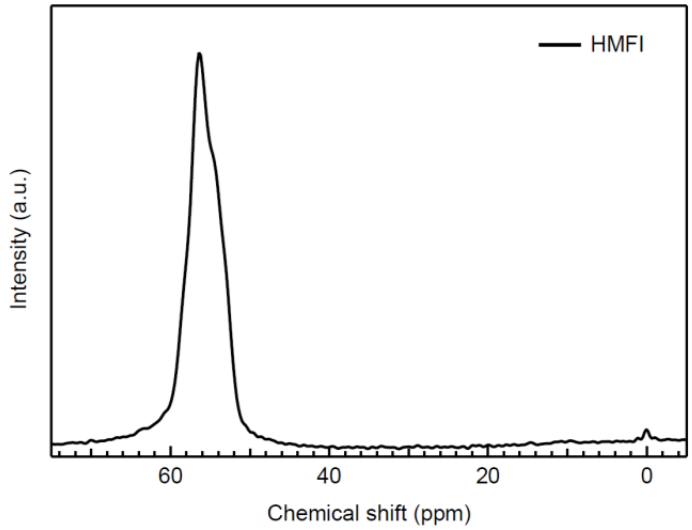
HMFI
Intensity (a.u.)
60
40
20
0
Chemical shift (ppm)

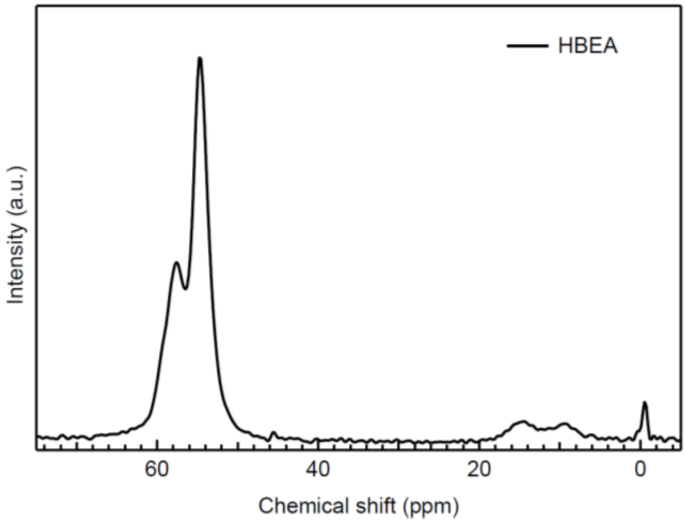
HBEA
Intensity (a.u.)
60
40
20
0
Chemical shift (ppm)

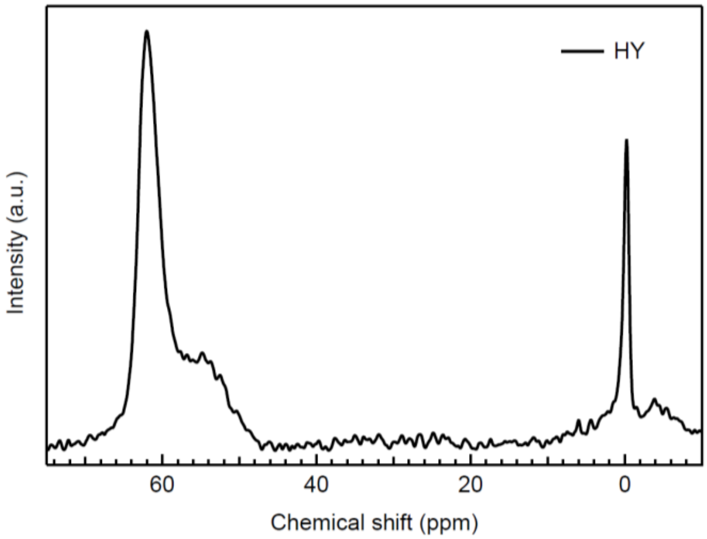
HY
Intensity (a.u.)
60
40
20
0
Chemical shift (ppm)

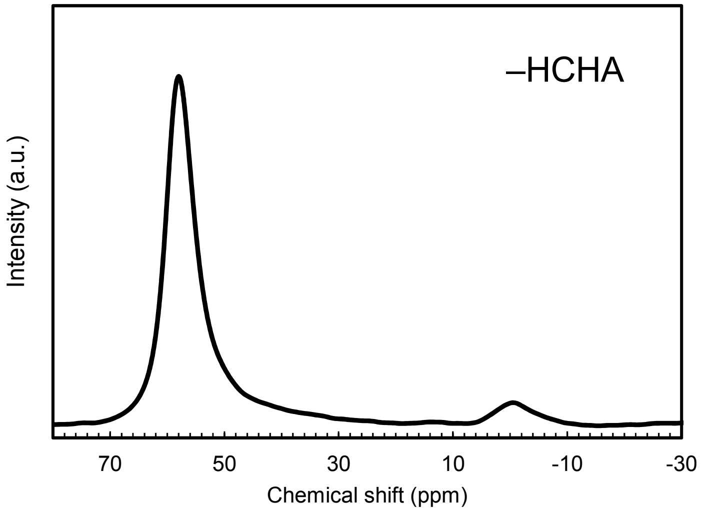

Supplementary Figure 13 | ^{27}Al MAS NMR spectra of the solid acids (from top to bottom: HMFI45, HBEA75, HY30, HCHA35). All spectra were measured on a 850 MHz spectrometer except for HCHA35 which was measured on a 500 MHz spectrometer which might lead to some quadrupole line broadening and invisible Al.

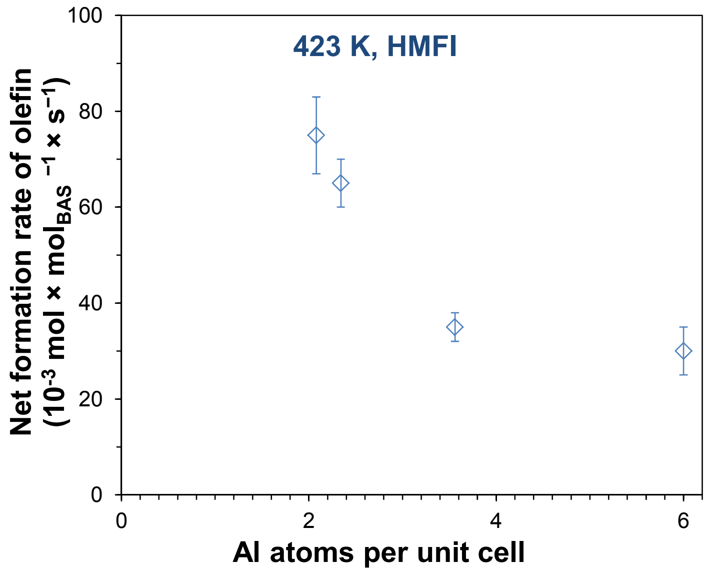

Supplementary Figure 14 | Net formation rates of olefin from aqueous phase cyclohexanol dehydration as a function of Al concentration in the MFI framework. The BAS concentrations used to normalize rates were determined from IR measurements of adsorbed pyridine (that desorbs at 423 K). The error bars in rates reflect the uncertainties based on linear regression fits of the conversion-time plots with 95% confidence level. The Al concentrations (Al atom per unit cell) were estimated based on ^{27}Al MAS NMR measurements. The average particle sizes are 0.5-1 μm for these MFI samples according to SEM measurements.

$$ROH(aq) + H^+(H_2O)_4(aq) \overset{K_{L,a}}{\rightleftharpoons} H^+(H_2O)_3(ROH)(aq) + H_2O(l)$$

$$H^+(H_2O)_3(ROH)(aq) \overset{K_{prot}}{\rightleftharpoons} (H_2O)_3(ROH_2{}^+)(aq)$$

$$(H_2O)_3(ROH_2{}^+)(aq) \underset{k_r}{\overset{k_f}{\rightleftharpoons}} (H_2O)_4(R^+)(aq) \quad with\ K_{C-O} = \frac{k_f}{k_r}$$

$$(H_2O)_4(R^+)(aq) \xrightarrow{k_{C\text{-}H}} R(-H) + H^+(H_2O)_4(aq)$$

Supplementary Figure 15 | A proposed sequence of steps within an E1-type mechanistic framework for aqueous phase dehydration of cyclohexanol catalyzed by hydronium ion in solution. Association of the alcohol with hydronium ion and the subsequent protonation is proposed to be sufficiently fast and quasi-equilibrated (a circle on top of a two-way arrow). The base in the deprotonation step (C–H bond cleavage) is H_2O in this scheme. The hydronium ion is represented as $H^+(H_2O)_4$(aq), the association complex as $H^+(H_2O)_3$ROH(aq), the olefin product as R(-H).

$$ROH(aq) + H^+(H_2O)_4(aq) \overset{K_{L,a}}{\rightleftharpoons} H^+(H_2O)_3(ROH)(aq) + H_2O(l)$$

$$H^+(H_2O)_3(ROH)(aq) \overset{K_{prot}}{\rightleftharpoons} (H_2O)_3(ROH_2{}^+)(aq)$$

$$(H_2O)_3(ROH_2{}^+)(aq) + H_2O\ (l) \xrightarrow{k_{rds}} R(-H) + H_2O(l) + H^+(H_2O)_4(aq)$$

Supplementary Figure 16 | A proposed sequence of steps within an E2-type mechanistic framework for aqueous phase dehydration of cyclohexanol catalyzed by hydronium ion in solution. Association of the alcohol with hydronium ion and the subsequent protonation is proposed to be sufficiently fast and quasi-equilibrated (a circle on top of a two-way arrow). The base in the rate determining step is H_2O in this scheme. The hydronium ion is represented as $H^+(H_2O)_4$(aq), the association complex as $H^+(H_2O)_3$ROH(aq), the olefin product as R(-H).

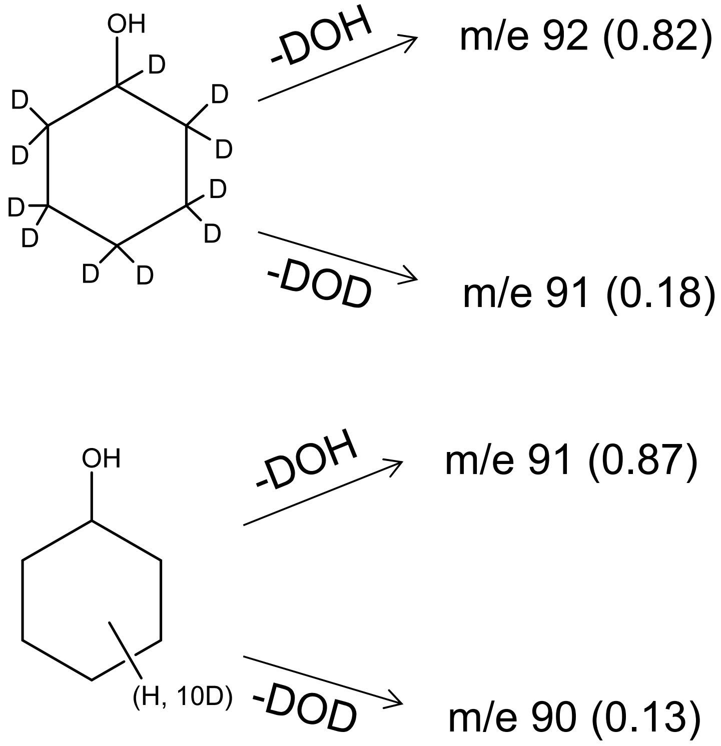

Supplementary Figure 17 | Fragmentation processes and their experimental probabilities (denoted as the values in the parentheses) for overall water loss. Data from ref.[4].

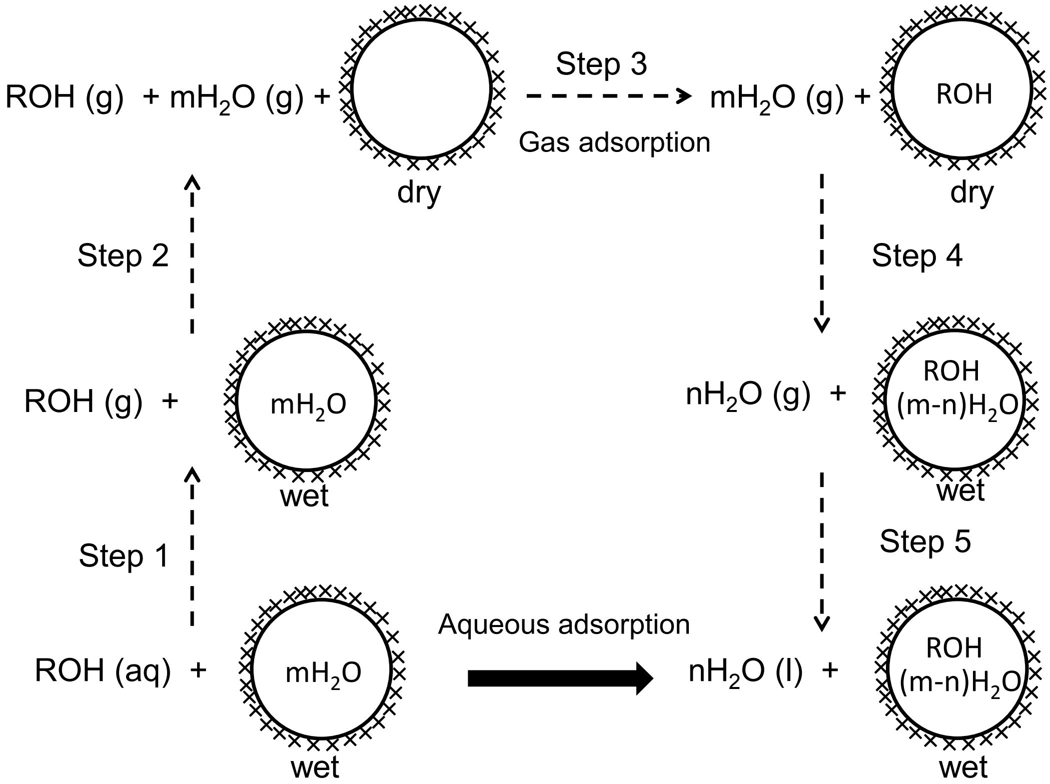

Supplementary Figure 18 | A thermochemical cycle that links gas phase and aqueous phase adsorption of cyclohexanol into zeolitic voids.

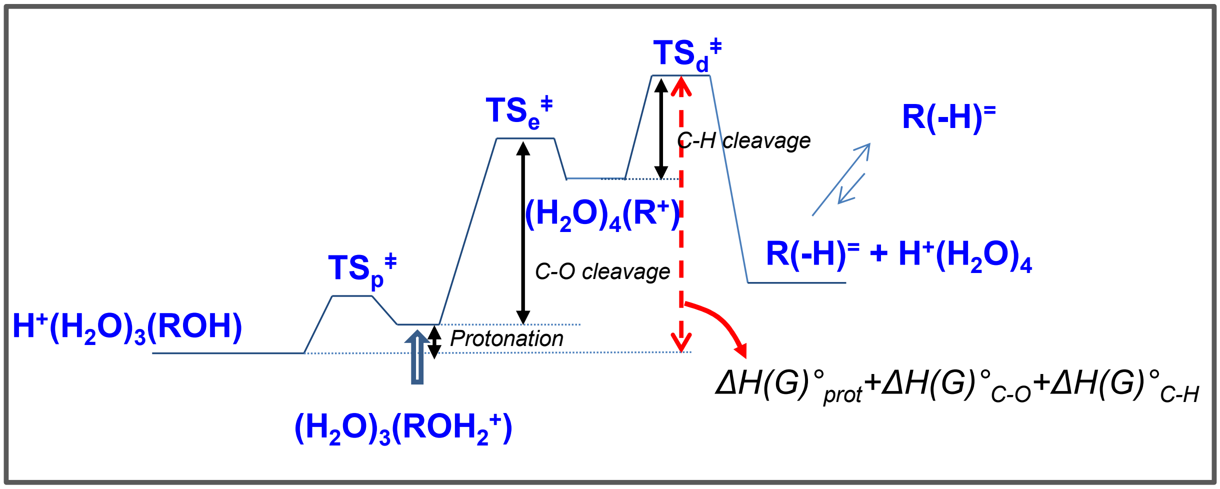

Supplementary Figure 19 | An illustrative free-energy diagram of alcohol (ROH) dehydration in aqueous phase, via an E1-type mechanism with kinetically relevant C–H bond cleavage. All intermediates and TSs (TS_p, protonation of alcohol; TS_e, C–O bond cleavage leading to elimination of water; TS_d, deprotonation of carbenium ion) are solvated.

Supplementary Note 1. General considerations and extended data

S1.1 A brief overview of mechanistic considerations

The intramolecular dehydration of mono-alcohols is Brønsted acid-catalyzed and is postulated to start with the quasi-equilibrated protonation of the alcoholic OH to form an alkoxonium ion, followed by elimination of water and formation of the olefin (in one or two elementary steps). Subsequent steps (e.g., olefin desorption, 1,2-hydride shift, rehydration, isomerization and C–C coupling) are irrelevant to the overall dehydration kinetics at low conversions and remote-from-equilibrium conditions. Classically, two major elimination pathways are operative: on the E1-type paths (Supplementary Fig. 11a and b), the protonated alcohol intermediate undergoes stepwise cleavage of the C_α–O and C_β–H bonds; alternatively, the E2-type path involves concerted scission of the C_α−O and C_β−H bonds in the protonated alcohol, forming olefin and water concurrently with proton transfer back to the acid catalyst (Supplementary Fig. 11c). Less common is a concerted pericyclic elimination, where intramolecular β-H transfer to the leaving water is concerted with C_α–O bond cleavage in a cyclic transition state (TS).

Any of the above situations are possible,[5] depending on the alcohol structure, nature/strength of the base, polarity of the reaction media and reaction temperature. In an aqueous solution without a base strong enough to abstract a hydron from the β-carbon and on solid surfaces with predominantly acidic properties, an E1cB mechanism is highly unlikely. These mechanistic aspects, despite being well documented in homogeneous acid-catalyzed dehydration, are examined for hydrated solid surfaces in water.

S1.2 Corrections for reverse reactions

Alcohol dehydration forming olefin and water is often a reversible reaction, for which the gas-phase thermochemistry is typically known. For instance, cyclohexanol dehydration to cyclohexene and water has a standard reaction enthalpy and entropy of 43.8 kJ mol^{-1} and 145.4 J mol^{-1} K^{-1}, respectively. In aqueous phase, however, the lack of thermodynamic data for the partitioning of reactant and products among different phases at elevated temperatures renders the direct assessment of reaction equilibrium constants and reaction quotients rather difficult. To circumvent this problem and determine the extent of the reverse olefin hydration reaction, a small quantity (5–10 mg) of a reduced Pd/Al_2O_3 catalyst (Pd dispersion: 11 %; immeasurable activity when used alone for dehydration at 413−473 K) was added to catalyze the rapid

hydrogenation of cyclohexene produced. By this means, the back reaction was essentially eliminated, leading to a predominant fraction (> 95%) of cyclohexane in the products. The turnover rates determined this way on the basis of cyclohexane formation are ca. 5–10% higher than those measured only with an acid catalyst (Supplementary Table 6); this difference is slightly beyond experimental uncertainties, indicating a small extent of back reaction.

However, as the olefin-hydrogenating function was not present inside the pores of the zeolites, this approach did not allow determining the relative extent of rehydration of cyclohexene while it diffuses out of the pore. A careful mass fragmentation pattern analysis of the isotopomeric composition of the recovered alcohol in $C_6D_{11}OH$–H_2O experiments (Supplementary Note 4) reveals that the rate of the back reaction was less than 10% of the forward reaction rate on BEA (~10% conversion), while olefin rehydration was found to occur at significant rates inside the pore of MFI (> 30% of forward rate at ~9% conversion). Thus, measured reaction rates at low conversions (< 10%) reflect the forward rates on all studied acids except for MFI where forward rates would be at least 30% higher than measured rates on account of the significant back reaction even at low conversions.

S1.3 Secondary pathways

C–C coupling was not observed on any zeolites at conversions lower than 50%; in contrast, a control experiment using cyclohexene added in quantities corresponding to 30% dehydration conversion already showed significant C–C bond formation (Supplementary Fig. 12). This difference suggests that the intraporous concentration of cyclohexene is low when that of cyclohexanol is relatively high, a result of competitive adsorption.

S1.4 Characterization results of solid acids

The textural (BET surface areas and micropore/mesopores volumes) and acidic properties of the studied solid acids, along with the used counterparts, are compiled in Supplementary Table 1. For zeolites, Si/Al ratios measured by AAS are also shown for the fresh samples. The mean crystallite sizes of commercial zeolites were previously characterized in a number of publications. The primary crystallite size of MFI45 (Clariant) is typically smaller than 100 nm, not exceeding 500 nm. BEA75 (Clariant) contains particles with rounded corners and average diameters of ~200−300 nm. FAU30 (Zeolyst) has an average crystal size of 0.4–0.6 μm. CHA35,

synthesized in-house, was characterized in this work by He ion microscopy to exhibit cuboid particles of ~2 μm.

The titration experiments using dilute aqueous pyridine and 2,6-dimethylpyridine (2,6-lutidine) solutions yielded estimates comparable to those from gas-phase infrared measurements using the pyridinium absorption band at ~1540 cm^{-1} (within a factor of 1.3; Supplementary Table 1).

^{27}Al MAS NMR spectra have been measured for zeolites (Supplementary Fig. 13). Note that with the exception of FAU, all the studied zeolites contain negligible to small concentrations of octahedral Al species (~0 ppm).

S1.5 Stability of solid acids in water during rate measurements

Solid acids have different hydrothermal stabilities in aqueous media, which represents a major challenge against rigorous rate measurements. Specifically, both textural and acidic properties of MFI and BEA zeolites remain hardly affected during reactions (i.e., with cyclohexanol present) on the time scale of up to 2 h (Supplementary Table 1). Recovered MFI and BEA catalysts showed unchanged activities in the consecutive run (Supplementary Fig. 1). In contrast, Y-zeolite (FAU) is known to undergo structural degradation in hot liquid water.[6,7] As a previous study[6] showed that the crystallinity, porosity and acidity of Y-zeolites (Si/Al = 14 and 41) started to change significantly beyond 1 h at 423–473 K in liquid water, we performed reactions for shorter periods of time (up to 1 h) on these two materials to minimize/negate possible corruption of measured kinetics by site losses and/or alteration in microenvironment along the reaction course. The linear increase in substrate conversion with time (Supplementary Fig. 2) suggests that active sites on FAU remain *functionally* intact within this time scale.

S1.6 Effect of the Si/Al ratio in MFI and BEA framework

Although changing the Si/Al ratio for a given zeolite framework is anticipated to affect the site-normalized activity (i.e., TOF) to some extent, our previous study, employing a series of HBEA zeolites with Si/Al ratios of 15–75 (i.e., 0.8–4.0 Al/u.c.), different concentrations of Si–O–Si connectivity defects, and negligible amounts of extra-framework Al species (EFAL), demonstrated that TOFs are nearly independent of the Si/Al ratio for aqueous-phase dehydration of cyclohexanol on HBEA.[8] The relatively weak dependence of rates (per BAS) on framework Al density and silanol defect concentration for BEA zeolites provides additional evidence that measured turnover rates in liquid water are not convoluted by coupled intracrystalline diffusion

phenomena of cyclohexanol (kinetic diameter ~0.6 nm) to active centers confined within HBEA channels. For HMFI zeolites where the concentration of EFAL also remained low, the TOFs (net olefin formation rates) decreased as the Si/Al ratio decreased from 45 to 15 (2.0–6.0 Al/u.c.; Supplementary Fig. 14). After correcting for a greater extent of back reaction with a higher BAS density in the MFI channel, the difference in the forward dehydration rate should be rather limited (a factor of ~2).

Given that Si/Al ratio affects the hydrophilicity/hydrophobicity of, and the spatial proximity of BAS[9] inside the intraporous environment,[10–12] we infer that neither of these two factors are considerably altered in the studied range of Si/Al ratio, or that they, at best, have limited impact on this reaction when operated within the zero-order kinetic regime (saturation of active sites for all zeolites except CHA) and when the nature of the active site remains unchanged. However, it is reasonable to anticipate that, for highly hydrophobic zeolites (e.g., those modified with organosilanes[13]) where water intrusion and association with the internal BAS is strongly impeded, the active site could significantly differ from intrazeolitic “hydronium ion” as in relatively hydrophilic zeolites (as used in this work), and might be better represented by framework-bound proton which was shown to catalyze the reaction with completely different energetics (e.g., in gas-phase or neat liquid alcohol phase).

Supplementary Note 2. Thermochemical analysis of adsorption measurements

When pores are fully occupied, the adsorption of cyclohexanol from dilute aqueous solution will result in displacement of intraporous water molecules out of the pores, which can be written as a reversible exchange process between the interfacial monolayer and the bulk phase:

$C_6H_{11}OH$ (aq) + nH_2O(ads) = $C_6H_{11}OH$ (ads) + nH_2O(aq)

For this process, its equilibrium constant K can be defined as:

$$K = \frac{\{H_2O_{aq}\}^n N_{C_6H_{11}OH_{ads}}}{{N_{H_2O_{ads}}}^n \{C_6H_{11}OH_{aq}\}} \quad (1)$$

where $\{H_2O_{aq}\}$ and $\{C_6H_{11}OH_{aq}\}$ correspond to the activities of water and cyclohexanol in solution, while $N_{H_2O_{ads}}$ and $N_{C_6H_{11}OH_{ads}}$ correspond to the mole fractions of water and cyclohexanol in the adsorbed phase, respectively. Since the surface or pore is fully covered under measurement conditions, $N_{H_2O_{ads}}$ and $N_{C_6H_{11}OH_{ads}}$ add up to unity. In deriving the entropy quantities from the measured K, activities of the adsorbed species, adsorbing species, and solvent were replaced by the mole fractions (note: using concentration standard state, 1 mol L^{-1}, for solution species does not change the adsorption entropy) and the non-ideality of the adsorption systems was neglected.

In an aqueous adsorption experiment (thick arrow) starting with cyclohexanol (ROH) in aqueous solution and a zeolite saturated with water inside the pore, ROH enters the zeolite pore and replaces a fraction (n moles relative to 1 mole of ROH) of H_2O (which diffuses out to the aqueous solution) in the pore. A thermochemical cycle (Supplementary Fig. 18) can be constructed to link adsorption of cyclohexanol into zeolites from aqueous solutions and from gas phase. There are equilibrium constants, some being unmeasurable, associated with each hypothetical step. Similarly, there are corresponding ΔH and ΔS for each step.

The cycle in Supplementary Fig. 18 is dissected into the following steps (dashed arrows):

(Step 1: K_1, ΔH_1, ΔS_1, standard states are 1 mol L^{-1} and 1 bar for solution and gas phase, respectively) This step starts with the initial state in the aqueous ROH adsorption. The aqueous ROH is brought to gas phase. The Henry's law constant (K_H) should be the appropriate parameter to describe the solvation of gas phase molecule by aqueous medium. K_H for cyclohexanol in water is available (170 mol L^{-1} bar^{-1}) at 293 K;[14] its temperature dependence,

however, is not reported. For a number of alcohols (C_2–C_4 aliphatic alcohols), the temperature dependences correspond to $-\Delta H$ = 53−65 kJ mol^{-1} for K_H.[15] Thus, we used 57 kJ mol^{-1} as the mean value of the temperature dependence for Step 1. The entropy associated with this step would be 152 J mol^{-1} K^{-1}. Enthalpy and entropy changes in this step are not zeolite-dependent properties.

(Step 2: K_2, ΔH_2, ΔS_2) Next, water in the zeolite pore desorbs and the zeolites are brought from a wet state (immersed in an aqueous solution) to a dry state. The enthalpy and entropy changes for the reverse of this step approximately equal the sum of adsorption enthalpy/entropy of water into zeolitic voids ($-m\times\Delta H/S_{ads,g,water}$), which is zeolite-dependent, and hydration enthalpy/entropy of external surfaces ($-\Delta H/S_{hydration,ext}$), which is supposedly similar for different zeolites studied in this work.

(Step 3: K_3, ΔH_3, ΔS_3, standard states are 1 bar and pore filling fraction = 1 for gas phase and adsorbed phase, respectively) This step corresponds to cyclohexanol adsorption from gas phase into zeolite pore. The adsorption enthalpies for gas phase cyclohexanol onto the MFI45, BEA75 and FAU30 samples were measured to be –90, –78 and –50 kJ mol^{-1}, respectively (details to be shown elsewhere); the concomitant entropy changes were –201, –160 and –75 J mol^{-1} K^{-1}. Detailed results will be shown in a following contribution.

(Step 4: K_4, ΔH_4, ΔS_4) In this step, the external surface of the zeolite is hydrated/wetted ($\Delta H/S_{hydration,ext}$) and a fraction of gaseous H_2O molecule ($(m-n)\times\Delta H/S_{ads,g,water}$) adsorbs inside the zeolite. The hydration enthalpy/entropy in this step fully cancels out the reverse process (“wet” zeolite to “dry” zeolite) in Step 2, while the adsorption of water partially cancels out the reverse process in Step 2. With the presence of co-adsorbed ROH, the adsorption strength and entropy of water could be somewhat different. As it is challenging to assess accurately and quantitatively the interaction between ROH and water in the pore, it is assumed that the adsorption (both enthalpy and entropy changes) of ROH and H_2O is not affected by each other.

(Step 5: K_5, ΔH_5, ΔS_5) This step fully recovers the end state in the aqueous ROH adsorption by converting n moles of water (g) from gas to liquid, with the corresponding enthalpy and entropy changes being condensation heat of n moles of water ($n\times\Delta H/S_{cond,water}$) and zeolite-independent. $\Delta H_{cond,water}$ is –44 kJ mol^{-1}, while $\Delta S_{cond,water}$ is –119 J mol^{-1} K^{-1}.

For a given zeolite, the following relations would then result from the above decomposition of the thermochemical cycle:

$$\Delta H_{ads,ROH,aq} = \Delta H_{ROH,aq\rightarrow g} - n\times\Delta H_{ads,water,g} + \Delta H_{ads,ROH,g} + n\times\Delta H_{cond,water} \quad (2)$$

$$\Delta S_{ads,ROH,aq} = \Delta S_{ROH,aq\rightarrow g} - n\times\Delta S_{ads,water,g} + \Delta S_{ads,ROH,g} + n\times\Delta S_{cond,water} \quad (3)$$

where $\Delta H/S_{ROH,aq\rightarrow g}$ are enthalpy/entropy changes for bringing cyclohexanol from aqueous solutions to gas phase; $\Delta H/S_{ads,ROH,aq}$ are enthalpy/entropy changes for adsorption of cyclohexanol from aqueous solutions into zeolite; $\Delta H/S_{ads,ROH,g}$ are enthalpy/entropy changes for adsorption of cyclohexanol from gas phase into zeolite; $\Delta H/S_{ads,water,g}$ are enthalpy/entropy changes for adsorption of H_2O from gas phase into zeolite; $\Delta H/S_{cond,water}$ are enthalpy/entropy changes for water condensation; n is the moles of water molecules replaced by 1 mole of cyclohexanol.

With the decreasing size of confines (MFI > BEA > FAU), $\Delta H/S_{ads,ROH,g,Z}$ becomes less negative, while $-n\times(\Delta H/S_{ads,H_2O,g,Z} - \Delta H/S_{cond,H_2O})$ is expected to become less positive or even become negative. Consequently, the enthalpy and entropy change for aqueous phase adsorption would turn positive when the sum of $\Delta H/S_{ROH,aq\rightarrow g}$ and $-n\times(\Delta H/S_{ads,H_2O,g,Z} - \Delta H/S_{cond,H_2O})$ overrides $-\Delta H/S_{ads,ROH,g,Z}$. Thus, the positive enthalpy and entropy change for adsorption on FAU (Table 1 in the main text) suggest that cyclohexanol is significantly less solvated in FAU zeolite (where there are also intraporous water and hydronium ions) than in aqueous solution.

Supplementary Note 3. Estimation of adsorption capacity under reaction conditions

The adsorption isotherms of cyclohexanol from aqueous solutions onto zeolites HMFI45, HBEA75 and HFAU30 (number denotes measured Si/Al ratio) have been measured at various temperatures (280–353 K). Langmuir-type adsorption model, as discussed in the main text, has been applied to fit these measured isotherms to obtain adsorption constant (K_{ads}) and saturation uptake (q_{max}) at each temperature. Detailed results will be reported in a subsequent publication. For all zeolites, the saturation uptake of cyclohexanol from aqueous solutions was remarkably lower than that measured from gas-phase adsorption (1.1, 2.2 and 2.3 mmol g^{-1} for MFI45, BEA75 and FAU30, respectively). This appears to reflect a significant amount of water adsorbed on these zeolites in contact with aqueous solutions. The saturation uptake of cyclohexanol increased by more than a factor of 2 from MFI to BEA, while the similar cyclohexanol uptakes on BEA and FAU likely stem from a higher fraction of volume inaccessible to cyclohexanol in FAU as well as a higher quantity of intraporous water in the more defective and hydrophilic FAU.

Next, we show how we determined adsorption capacity under reaction conditions. It was found that the saturation uptake decreased as adsorption temperature increased (Supplementary Table 4). This decrease in the saturation uptake with increasing adsorption temperature stems from the decrease in density of the adsorbate phase in the micropore (like thermal expansion of a liquid) as a function of temperature. The temperature dependence takes the form:

$$\frac{1}{q_{max}}\frac{dq_{max}}{dT} = -\delta \tag{4}$$

where δ is the temperature coefficient of expansion.[16]

Plotting measured/regressed saturation adsorption capacity at different temperatures as a function of temperature yielded a slope (–δ) of –0.0032, –0.0032 and –0.0012 K^{-1} for HMFI, HBEA and HFAU zeolite samples. Having extrapolating these experimentally determined q_{max} and K_{ads} to reaction temperatures using the same temperature dependence as determined between 280 and 353 K, we found that the saturation uptake of cyclohexanol would decrease from 0.40 to 0.35, from 1.05 to 0.92, and from 1.26 to 1.20 mmol g^{-1}, for HMFI, HBEA and HFAU, respectively,

with temperature increasing from 433 to 473 K. Assuming that the remaining micropore volume (total V_{micro} = 0.12, 0.20 and 0.26 cm^3 g^{-1}, respectively, for HMFI, HBEA and HFAU) is filled by adsorbed water (with density 0.90 at reaction temperature), the uptake of water in the pore would be 3.8±0.1, 4.0±0.2 and 6.2±0.4 mmol g^{-1}, respectively for HMFI, HBEA and HFAU (compared with 3.0, 1.8 and 5.9 $mmol_{H2O}$ g^{-1} at room temperature, correspondingly) at 433–473 K. Independent thermogravimetric analysis (TGA) shows that 1 g of HMFI sample stored under ambient conditions (100% RH) contains 0.042 g water, corresponding to 2.2 $mmol_{H2O}$ g^{-1}.

Supplementary Note 4. Mass fragmentation pattern analyses

For aqueous phase dehydration of perdeuterated cyclohexanol (which forms $C_6D_{11}OH$ upon exchange with the solvent, H_2O) that occurs along an E1-type path, the carbenium ion intermediate ($C_6D_{11}^+$) either gets deprotonated to form C_6D_{10} or reacts with H_2O to re-form alcohol without scrambling H/D at C–D bonds. Only via back reaction of olefin rehydration (C_6D_{10} + H_2O) can H/D scrambling occur at ring positions. Thus, in principle, the analysis of mass fragmentation pattern for the recovered $C_6D_{11}OH$ (dissolved in dichloromethane) should provide insight into the extent of back reaction on different catalysts at the studied conversion levels.

The mass spectrum of cyclohexanol has been the subject of numerous studies. For $C_6H_{11}OH$, the fragment ions of particular interest in the 70 eV spectrum (Supplementary Fig. 9(a2)) and the processes thought to be predominantly responsible for their formation are: (1) m/z 82, [M – HOH]; (2) m/z 57, [M – C_3H_7]; (3) m/z 67, [(M - HOH) – CH_3]; (4) m/z 72, [M – C_2H_4] and (5) m/z 71, [M – C_2H_5]. The water loss fragment, [M – HOH], was used in our analysis, as the fragmentation mechanism for this water loss process is relatively well understood.[4]

In the case of $C_6H_{11}OH$ (molecular weight 111), the m/z 82 fragment ion can lose one more H to yield m/z 81, which is present at 10% of the intensity of the m/z 82 peak. In the case of $C_6D_{11}OH$ (molecular weight 111, abbreviated as M-d_{11}), the loss of DOH leads to the dominant water loss fragment at m/z = 92 (C_6D_{10}). Despite that C_6D_{10} can only lose D to form m/z = 90, the m/z 91 ion (C_6D_9H) is always formed even without reactions, due to: i) scrambling of the H in the hydroxyl into ring positions during GC-MS analysis; 2) initial presence of $C_6D_{10}HOH$ (abbreviated as M-d_{10}) in the starting isotopomer reactant, which can lose DOH to form C_6D_9H. While m/z 92 is almost exclusively from $[M\text{-}d_{11}\text{–DOH}]^+$ ($[M\text{-}d_{10}\text{–HOH}]^+$ not considered because of very low probability), m/z 91 contains contributions from $[M\text{-}d_{10}\text{–HOD}]^+$ and $[M\text{-}d_{11}\text{–DOD}]^+$, and m/z 90 contains contributions from $[M\text{-}d_{10}\text{–DOD}]^+$, $[M\text{-}d_{11}\text{–HOD–D}]^+$ and $[M\text{-}d_{11}\text{–DOD–H}]^+$.

Supplementary Table 7 compiles the normalized single ion intensities for m/z = 90, 91 and 92. Note that all single ion intensities have been corrected for natural abundance ^{13}C (e.g., fragment group m/z 90, 91 and 92). The contribution of individual fragmentation processes to overall

water loss has been studied thoroughly.[4] The isotopic reactant, after complete exchange of the OD group with H_2O, contained M-d_{11} and M-d_{10} (combined for total D content > 98%). For the M-d_{11} parent ion (m/z 111, see Supplementary Fig. 9) to lose HOD (form m/z 92) and DOD (form m/z 91), the measured probabilities are 0.82 and 0.18 (Supplementary Fig. 17), respectively; values are taken from the normalized intensity data for cyclohexanol-OD, assuming no isotope effects. Subtracting the contribution of $[\text{M-d}_{11}\text{–DOD}]^+$ to the m/z 91 ion, which is 0.22 of the intensity of the m/z 92 ion, the $[\text{M-d}_{10}\text{–HOD}]^+$ accounts for 0.14 of the intensity of m/z 92. For the M-d_{10} ($C_6D_{10}HOH$) isotopomer to lose HOD (form m/z 91) and DOD (form m/z 90), the probabilities are estimated to be 0.87 and 0.13, respectively (Supplementary Fig. 17), on the basis of the normalized intensity data for 3,3,5,5-d_4-cyclohexanol. Note that D atoms in 2-, 4- and 6-positions do not induce the DOD loss pathway and that the appearance of H at any of the 3- and 5-positions reduces the probability of DOD loss from M-d_{10}. Therefore, the above estimated ratio for DOD and HOD losses from M-d_{10} (0.13/0.87 = 0.15) represents the theoretical maximum. Subtracting the contribution of $[\text{M-d}_{10}\text{–DOD}]^+$ to the m/z 90 ion, which is 0.021 (i.e., 0.14*0.15) of the intensity of the m/z 92 ion, the $[\text{M-d}_{11}\text{–HOD–D}]^+$ and $[\text{M-d}_{11}\text{–DOD–H}]^+$ combines for 0.08 of the intensity of m/z 92.

The analysis presented above indicates that the reactant is a mixture of 88% M-d_{11} and 12% M-d_{10}. Applying the same quantitative analysis, we found that the recovered alcohol contained 82% M-d_{11} and 18% M-d_{10} for MFI-catalyzed reaction (at conversion 9%), 87% M-d_{11} and 13% M-d_{10} for BEA-catalyzed reaction (conversion 11%), and 85% M-d_{11} and 15% M-d_{10} for H_3PO_4-catalyzed reaction (conversion 18%). Note that intensities for fragment ions of other isotopomers (e.g., m/z = 89 which contains contribution from $[C_6H_2D_9OH\text{–DOD}]^+$) remain much lower than M-d_{10} and M-d_{11} in all cases.

As the reverse olefin hydration events (with a rate of r_b) occur during dehydration (r_f), the H content in the recovered alcohol increases. From the isotopomer compositions before and after reaction, the extent of back reaction (r_b/r_f) can be calculated. For instance, we started with 0.1 mol L^{-1} reactant, which contains 0.088 and 0.012 mol L^{-1} for $C_6D_{11}OH$ and $C_6D_{10}HOH$, respectively. If both isotopomers reacted at the same rate, which should be the case due to similarly high D contents and thus little isotope effect, the final concentrations for $C_6D_{11}OH$ and $C_6D_{10}HOH$ in a recovered reaction mixture, at 9% conversion on MFI, should be 0.075 and

0.016 mol L^{-1}, respectively. The rate of back reaction multiplied by reaction time (1200 s) should equal to the sum of the net gain in concentration of M-d_{10} (0.016–0.012 = 4.0×10^{-3} mol L^{-1}) after reaction and the consumed M-d_{10} due to dehydration, the latter being at least 0.012 (initial M-d_{10} concentration)*0.09 (overall conversion) = 1.1×10^{-3} mol L^{-1}. With the net rate of overall dehydration (M-d_{11} and M-d_{10}) being 7.5×10^{-6} mol L^{-1} s^{-1} and the rate of back reaction (r_b) being ~4.4×10^{-6} mol L^{-1} s^{-1}, the forward dehydration rate (r_f) would be 1.2×10^{-5} mol L^{-1} s^{-1}. Thus, the reverse reaction already occurs significantly (r_b/r_f = 0.37) even at conversion of 9% on MFI. Similarly, for BEA- and H_3PO_4-catalyzed reactions, r_b/r_f = 0.10 (conversion 11%) and 0.13 (conversion 18%), respectively.

Supplementary Note 5. Derivations of rate expressions for different reaction pathways

In homogeneous acid-catalyzed alcohol dehydration in water, we suggest that the reaction starts with the association of hydronium ion (active site) with alcohol, which is effectively a displacement reaction of a H_2O molecule by an alcohol in the first solvation shell of the hydronium ion (equation (5)).

$$ROH(aq) + H^+(H_2O)_4(aq) \leftrightarrows H^+(H_2O)_3(ROH)(aq) + H_2O(l) \quad (5)$$

Under reaction conditions, this association step is considered rapid enough to be treated as quasi-equilibrated, with a thermodynamic constant $K_{L,a}$ (where the subscript "L" stands for the liquid phase, and "a" stands for association). For simplicity, we represent the hydronium ion as $H^+(H_2O)_4(aq)$ and the association complex as $H^+(H_2O)_3ROH(aq)$ here, while acknowledging that the number of water molecules is somewhat arbitrary and the structure of this complex is also vague. The extent of proton transfer from water cluster to ROH is not specified, either. However, these microscopic details do not affect derivations of the kinetic expressions below. We use concentration terms instead of activities for solution species in dilute systems, assuming activity coefficients for the solution species are unity.

For equation (5), i.e., association of cyclohexanol with hydronium ion, by letting the initial proton concentration be $[H^+]_0$, we have:

$$\frac{[H_2O(l)][H^+(H_2O)_3(ROH)(aq)]}{[ROH(aq)]([H^+]_0 - [H^+(H_2O)_3(ROH)(aq)])} = K_{L,a} \quad (6)$$

Solving the above equation gives:

$$\frac{[H^+(H_2O)_3(ROH)(aq)]}{[H^+]_0} = \frac{K_{L,a}\frac{[ROH(aq)]}{[H_2O(l)]}}{1 + K_{L,a}\frac{[ROH(aq)]}{[H_2O(l)]}} \quad (7)$$

The next step, proton transfer from water cluster to ROH, is required to weaken the C–O bond and prepare the intermediate for cleavage. For this rapid step, we have:

$$\frac{[(H_2O)_3(ROH_2{}^+)(aq)]}{[H^+(H_2O)_3(ROH)(aq)]} = K_{prot} \quad (8)$$

Thus,

$$\frac{[(H_2O)_3(ROH_2{}^+)(aq)]}{[H^+]_0} = \frac{K_{prot}}{1+K_{prot}} \frac{K_{L,a}\frac{[ROH(aq)]}{[H_2O(l)]}}{1+K_{L,a}\frac{[ROH(aq)]}{[H_2O(l)]}} \tag{9}$$

Mechanistic considerations diverge after the protonation step. For Supplementary Fig. 11(a) and (b), two subsets of classical stepwise E1-type mechanism, by applying steady-state assumption to the solvated carbenium-ion intermediate, $(H_2O)_4(R^+)(aq)$, we have (refer to Supplementary Fig. 15 for the meaning of rate and equilibrium constants for individual elementary steps):

$$k_f[H^+(H_2O)_3(ROH)(aq)] - k_r[(H_2O)_4(R^+)(aq)] = k_{C-H}[(H_2O)_4(R^+)(aq)] \tag{10}$$

$$[(H_2O)_4(R^+)(aq)] = \frac{k_f[H^+(H_2O)_3(ROH)(aq)]}{k_r+k_{C-H}} \tag{11}$$

The expression for TOF is (equal to that of the fourth step):

$$TOF_L = \frac{k_{C-H}[(H_2O)_4(R^+)(aq)]}{[H^+]_0} \tag{12}$$

Replacing the term for $[(H_2O)_4(R^+)(aq)]$, we have:

$$TOF_L = \frac{k_f k_{C-H}}{k_r+k_{C-H}} \frac{K_{prot}}{1+K_{prot}} \frac{K_{L,a}\frac{[ROH(aq)]}{[H_2O(l)]}}{1+K_{L,a}\frac{[ROH(aq)]}{[H_2O(l)]}} \tag{13}$$

We consider that in dilute acid solutions, H_2O is the most likely base, when there is no external base added, that is abundantly present. For zeolites, intraporous water is most likely the base that deprotonates the carbenium ion intermediate. Note that showing water as the product in C–O bond cleavage and as the base in the deprotonation step, as shown below (cf. Supplementary Fig. 15), does not change the TOF expression.

$$(H_2O)_3(ROH_2{}^+)(aq) \underset{k_r}{\overset{k_f}{\rightleftharpoons}} (H_2O)_3(R^+)(aq) + H_2O$$

$$(H_2O)_3(R^+)(aq) + H_2O \xrightarrow{k_{C\text{-}H}} R(-H) + H^+(H_2O)_4(aq)$$

Two extreme scenarios exist for E1-type mechanisms. In one, i.e., Supplementary Fig. 11(a), the microscopic reverse of C–O bond cleavage has a much higher free energy barrier than the deprotonation step such that $k_{C–H} >> k_r$, the TOF expression is simplified to:

$$TOF_L = \frac{k_f K_{prot}}{1+K_{prot}} \frac{K_{L,a}\frac{[ROH(aq)]}{[H_2O(l)]}}{1+K_{L,a}\frac{[ROH(aq)]}{[H_2O(l)]}} \quad (14)$$

The same rate expression would arise if the C–O bond cleavage were assumed to be irreversible and rate-determining. In this case, only secondary KIE is anticipated as none of the equilibrium and kinetic constants relate to a step where C–H bond is formed or cleaved.

At the other extreme, $k_{C-H} << k_r$, the TOF expression is simplified to:

$$TOF_L = \frac{k_{C-H} K_{C-O} K_{prot}}{1+K_{prot}} \frac{K_{L,a}\frac{[ROH(aq)]}{[H_2O(l)]}}{1+K_{L,a}\frac{[ROH(aq)]}{[H_2O(l)]}} \quad (15)$$

Provided that the TS for C–H bond cleavage (k_{C-H}) occurs late (product-like) along the reaction coordinate, primary KIE is anticipated in this case.

For a classical concerted E2-type path, i.e., Supplementary Fig. 11(c), the expression for TOF is (refer to Supplementary Fig. 15 for the meaning of rate and equilibrium constants for individual elementary steps):

$$TOF_L = \frac{k_{rds}[H^+(H_2O)_3(ROH)(aq)][H_2O(l)]}{[H^+]_0} \quad (16)$$

Replacing the term for $[H^+(H_2O)_3(ROH)(aq)]$, we have:

$$TOF_L = \frac{k_{rds}[H_2O(l)]K_{prot}}{1+K_{prot}} \frac{K_{L,a}\frac{[ROH(aq)]}{[H_2O(l)]}}{1+K_{L,a}\frac{[ROH(aq)]}{[H_2O(l)]}} \quad (17)$$

Note that the $[C_6H_{11}OH]$ term in rate expressions in Supplementary Fig. 11 corresponds to the association complex. So the equations derived above are identical to those shown in the main text.

It has been demonstrated from isotope experiments (see main text) that the prevalent dehydration mechanism is of predominant E1 character with the C_β–H bond cleavage as the kinetically relevant step, for aqueous phase dehydration of cyclohexanol catalyzed by both dilute homogeneous acids and acidic zeolites. Therefore, equation (15) would be the appropriate rate expression.

For homogeneous acid catalyzed dehydration, TOF ratios at different alcohol concentrations can be used to determine $K_{L,a}$ (equation (18)). The results are shown in Supplementary Table 8 of our previous work[2] and discussed in Supplementary Note 8.

$$\frac{TOF_{L,1}}{TOF_{L,2}} = \frac{\theta_{L,a,1}}{\theta_{L,a,2}} = \frac{\frac{K_{L,a}\frac{[ROH(aq)]_1}{[H_2O(l)]_1}}{1+K_{L,a}\frac{[ROH(aq)]_1}{[H_2O(l)]_1}}}{\frac{K_{L,a}\frac{[ROH(aq)]_2}{[H_2O(l)]_2}}{1+K_{L,a}\frac{[ROH(aq)]_2}{[H_2O(l)]_2}}} \quad (18)$$

Supplementary Note 6. Analysis of kinetic isotope effects

For dehydration of cyclohexanol ($C_6H_{11}OH$ and $C_6D_{11}OH$), the measured isotope effects (IEs) on the reaction rates reflect the effects of H/D identity on the individual rate and equilibrium constants, as shown below:

$$\frac{r_H}{r_D} = \frac{k_{3,H}K_{2,H}K_{1,H}}{k_{3,D}K_{2,D}K_{1,D}} \times \frac{1+k_{3,D}/k_{-2,D}}{1+k_{3,H}/k_{-2,H}} \times \frac{[C_6H_{11}\mathrm{OH}]_a}{[C_6D_{11}\mathrm{OH}]_a} \quad (19)$$

where $K_{1,H(D)}$ is the equilibrium constant for protonation of $C_6H_{11}OH$ ($C_6D_{11}OH$), $K_{2,H(D)}$ is the equilibrium constant for C–O bond cleavage in the protonated $C_6H_{11}OH$ ($C_6D_{11}OH$) (forming nondeuterated (perdeuterated) carbenium ion intermediate), $k_{-2,H(D)}$ is the rate constant for the C–O bond recombination between H_2O and the nondeuterated (perdeuterated) carbenium ion intermediate, and $k_{3,H(D)}$ is the rate constant for the C_β–H(D) bond cleavage in the nondeuterated (perdeuterated) carbenium ion intermediate. $[C_6H_{11}OH]_a$ and $[C_6D_{11}OH]_a$ should not be different, as alcohol-hydronium ion association ($K_{L,a}$) is not anticipated to change with H/D isotopic substitution at C–H bonds. There should be only a negligible effect of H/D isotopic substitution on the equilibrium constant K_1 for protonation of $C_6H_{11}OH$ or $C_6D_{11}OH$, as isotopic substitution should also hardly affect the thermodynamics of protonation of alcohol by hydronium ions.

According to Lowry and Richardson,[17] for a step involving the re-hybridization of α carbon from sp^3 to sp^2 in the TS, the IE value can be estimated by the following equation for K_2:[17]

$$\frac{K_{2,H}}{K_{2,D}} = \exp\left(-\frac{0.1865}{T}(\nu_P - \nu_R)\right) \quad (20)$$

where υ_P is the vibrational frequency of an C–H bond of the product state (carbenium ion) and the υ_R is the vibrational frequency of the corresponding C–H bond of the reactant state (protonated alcohol). For sp^3 hybridization on the α carbon of the protonated alcohol and sp^2 hybridization on α carbon of the carbenium ion, the υ_R is ~1350 cm^{-1} and the υ_P is ~800 cm^{-1}, so the estimated KIE value is ca. 1.25 at 433–473 K.

The KIE value can be estimated for an elementary step involving the complete cleavage of C–H bond by the following equation:

$$\frac{k_{3,H}}{k_{3,D}} = \exp\left(\frac{0.1865}{T}\nu_H\right) \quad (21)$$

where T is the absolute temperature and υ_H is the vibrational frequency of the C–H bond (~2985 cm^{-1}). Thus, the estimated KIE value involving the cleavage of a C–H bond is 3.2–3.6 at 433–473 K. This value is often attenuated from its theoretical maximum (in the absence of tunneling effect) as the C–H bond is often not fully broken at the TS.

The measured IEs (Table 1 in the main text) are somewhat smaller than the theoretical maximum (3.8–4.5). This may be explained by a less than fully broken C–H bond at the TS in the deprotonation step of the carbenium ion intermediate (TS3 in Supplementary Fig. 11b). In addition, we note that $\frac{1+k_{3,D}/k_{-2,D}}{1+k_{3,H}/k_{-2,H}}$ should be strictly smaller than 1, because $k_{3,D} < k_{3,H}$ while $k_{-2,D} \approx k_{-2,H}$. The ratio of k_3/k_{-2} reflects the free energy difference between TS3 and TS2, i.e., transition states for deprotonation of carbenium ion and for C–O bond cleavage of protonated alcohol, respectively.

Next, we consider three representative scenarios.

1) if $k_{3,H} = 0.1\ k_{-2,H}$ (the subscript "H" means nondeuterated reactant), the free energy difference between TS3 and TS2 ($\Delta G^{\circ}_{TS3,H}$–$\Delta G^{\circ}_{TS2,H}$) would be +8 kJ mol^{-1} and $\frac{1+k_{3,D}/k_{-2,D}}{1+k_{3,H}/k_{-2,H}}$ would be close to 1; in this case (or more generally, $k_{3,H} << k_{-2,H}$), the measured IE has to be reconciled by less complete C–H bond cleavage at TS3.

2) if $k_{3,H} = k_{-2,H}$, $\Delta G^{\circ}_{TS3,H}$–$\Delta G^{\circ}_{TS2,H}$ would be zero. Since $k_{3,H}/k_{3,D}$ is 3.2–3.6 (full C–H bond breaking at TS3) and $k_{-2,H} \approx k_{-2,D}$, $\frac{1+k_{3,D}/k_{-2,D}}{1+k_{3,H}/k_{-2,H}}$ would be 0.64. The overall IE predicted from the above theoretical analysis would be 2.9.

3) if $k_{3,H} = 5k_{-2,H}$, $\Delta G^{\circ}_{TS3,H}$–$\Delta G^{\circ}_{TS2,H}$ would be -5.8 kJ mol^{-1}, and $\frac{1+k_{3,D}/k_{-2,D}}{1+k_{3,H}/k_{-2,H}}$ would be 0.40. The overall IE would be 1.8.

Based on the measured KIEs, k_3 (C_β–H bond cleavage) should be smaller than or comparable to, but cannot be considerably greater than, k_{-2} (C_α–O bond recombination). Conceptually, the rate of olefin formation (r_o) relative to that of ^{18}O-scrambling (r_s) can be taken as a measure of the upper bound of $\frac{k_3}{k_{-2}}$, given that H_2O is the base to deprotonates the carbocationic intermediate. According to Table 3 (main text), r_o and r_s are comparable within a factor of 2, without correcting

for the extent of back reaction of olefin and some attack of $H_2{}^{16}O$ (present as impurity and also formed during reaction) on the cyclohexyl cation. In turn, k_3 should also be comparable to, or smaller than k_{-2}, which would lead to overall KIEs of > 2.8.

Taken together, the foregoing analyses clearly demonstrate that $k_{3,H}$ is smaller than, or comparable to, $k_{-2,H}$. It can be further deduced that when k_3 is much smaller than k_{-2}, the measured activation energy or free energy barrier is equal to the enthalpy/free energy change from the alcohol-hydronium ion association complex to the deprotonation TS (Supplementary Fig. 19).

It is also important to note that in the presence of severe intrazeolitic diffusion limitations, observed H/D KIE values would be proportional to the square root of the intrinsic KIE, which would then become smaller than 2, inconsistent with the measured values. External diffusion limitations would lead to insignificant KIEs.

Supplementary Note 7. Thoughts on rational design for acid catalysts for dehydration of cyclic alcohols in aqueous phase

In this section, we provide additional remarks on the hydronium-ion catalyzed dehydration.

First, we note that in aqueous solutions, homogeneous acids with different pK_a's do not show differences in the catalytic activity for alcohol dehydration on an active site (hydronium ion) basis. This was shown for cyclohexanol in the present work, but was found to be the case also for alkyl substituted cyclohexanol (to be shown in a subsequent contribution). We believe the conclusion is even more general to other substrates, as long as the reaction is hydronium ion catalyzed. Noteworthily, Mellmer et al. recently reported that the TOF of xylose dehydration in aqueous phase was dependent on the pK_a of a given homogeneous acid.[18] However, taking into account the dissociation constants of the weak acids at reaction temperatures (e.g., H_3PO_4, 448 K), the true TOF (normalized to hydronium ion concentration) was actually independent of the chemical identity of the homogeneous acid (strong or weak).

While the representation of hydronium-ion-type active site seems appropriate for relatively hydrophilic zeolites in aqueous phase, it may not apply to cases where intraporous water is present at much lower concentrations. The absence or very low concentrations of intraporous water has several potential consequences. First, with intraporous water sparsely present, the framework-bound proton has a higher tendency to remain as the prevalent form of BAS and exhibit a greater potential to protonate alcohol reactants, favoring the pre-equilibrium towards protonated alcohol. Second, as the intraporous concentration of water decreases, that of alcohol may increase. Due to the first two consequences, i.e., the increased alcohol concentration in the pore and the more pronounced alcohol protonation, the dominant form of adsorbed alcohol could shift from monomer to dimer, thereby opening another reaction path to olefin.[19] Finally, with the disappearance of additional solvation of intrazeolitic intermediates and transition states (TS) by water, the activation barriers and entropies would change accordingly.

HBEA zeolites catalyze cyclohexanol dehydration with TOFs several times higher in neat alcohol phase than in aqueous solutions (to be shown in another publication). If this rate enhancement reflects the intrinsic behavior of a zeolitic proton or a small hydronium ion complex (as opposed to a large hydronium ion complex as explored in this work), it would seem

beneficial, as a step forward, to devise synthetic strategies to contain BAS within reaction environments protected from liquid water. In the context of aqueous phase reactions, such microenvironment-engineering strategies include synthesis of zeolites in low-defect forms[18] and post-synthetic surface hydrophobization[20,21] or defect healing,[22] which have been shown to also efficiently enhance tolerance of zeolitic materials against hot liquid water.

For aqueous phase dehydration of other cyclic alcohols (alkylcyclohexanols), microporous zeolites (except for the small-pore zeolite, CHA) are also more active catalysts than homogeneous acids and mesoporous solid acids. In general, MFI is the most active zeolite catalyst on an active-site basis. The peculiarity of MFI stems from the closely similar dimensions of the pore and the solute molecule (cyclic alcohols, ~ 0.6 nm). Towards the formation of the kinetically relevant TS, the pore confines could decrease both the enthalpy and the entropy of the TS relative to the adsorbed alcohol. As a consequence, it is always the enthalpy-entropy compensation induced by the pore constraints, or in other words, the resultant Gibbs free energy barrier (Table 3 in the main text), that dictates the catalytic performance. As a result of the unfavorable activation entropy, 2-methylcyclohexanol dehydration becomes less active on MFI than on BEA at higher temperatures (not shown in this work). A comprehensive and more quantitative evaluation of ring-substituent effects on the reaction mechanism and energetics, as well as the underlying molecular-level origins of these effects, is to be reported in another contribution.

Supplementary Note 8. Calculation of association equilibrium constant for the alcohol-hydronium ion complex

Using measured turnover rates for homogeneous acid-catalyzed dehydration as a function of cyclohexanol concentration, the $K_{L,a}$ and k_{rds} were determined. The results were shown in the Supplementary Table 8 of our previous work.[2] The enthalpy and entropy changes determined from the Van't Hoff plot of the determined $K_{L,a}$ were found to be -3 kJ mol^{-1} and 24 J mol^{-1} K^{-1}, respectively.[2] At [ROH] = 0.32 M and [H_3O^+] = 3–4×10^{-3} M, 15–19% of the hydronium ions are associated with cyclohexanol, while greater extents of association are seen at higher alcohol concentrations, resulting in the observed fractional order. Importantly, we remark that nearly complete alcohol-hydronium ion association in aqueous solutions would be achieved only by increasing alcohol concentrations to levels (e.g., at > 5 M cyclohexanol) that would promote side reactions (ether formation and olefin oligomerization) and deactivation and lead to separate phases (solubility of cyclohexanol is ~10 g/100 mL water at 443 K according to our measurements) and ambiguity in the nature of the solvated active site.

In addition, we note that corrections for volume expansion and vaporization (water and cyclohexanol) have been made to obtain more accurate estimates for the actual [ROH]/[H_2O] ratio under reaction conditions. The extent of volume expansion is calculated based on the decrease of density of water at 160–200 °C. For the estimation of cyclohexanol vapor in the headspace, Henry's law constants (K_H) were first determined at 160 and 200 °C; K_H at 170–190 °C were obtained from interpolation using room temperature value (NIST) and the two measured values. After these corrections, the actual [ROH]/[H_2O] ratios at 160–200 °C for the 3.1 wt% and 9.1 wt% cyclohexanol solutions are 5.3–5.6×10^{-3} (vs. 5.8×10^{-3} at r.t.) and 1.5–1.6×10^{-2} (vs. 1.6×10^{-3} at r.t.), respectively. So these corrections tend to be very small.

These data have been presented and discussed in our recent work.[2]

Supplementary Methods

Extended description of experimental methods, theoretical calculations and error analysis of intrinsic activation parameters

Chemicals. Cyclohexanol (ReagentPlus®, 99%), cyclohexene (99%), 2-methylcyclohexanol (99%, mixture of cis and trans), 4-methylcyclohexanol (98%, mixture of cis and trans), pyridine (99.8%), 2,6-lutidine (98%), 1,3–dimethoxybenzene (99%), 1,3,5-trioxane (≥99%), dichloromethane (HPLC grade), sodium sulfate (99%, anhydrous) and cyclohexanol-d_{12} (98–99 atom% D; containing a small but undetermined fraction of $C_6D_{10}HOD$) were purchased from Sigma–Aldrich and used as received without further purification. Water ($H_2{}^{16}O$) was from the ultrapure (resistivity 18.2 MΩ cm) water dispenser system. $H_2{}^{18}O$ (97% isotope purity) was purchased from Cambridge Isotope Laboratories.

Catalysts. In addition to those described in the main text, several other HZSM-5 samples were prepared either by thermally treating (1 K min^{-1} ramp to 723 K and hold for 6 h) NH_4-ZSM-5 samples (Zeolyst, CBV3024E, CBV8014, and CBV28014, with Si/Al = 15, 40 and 140, respectively) in air (Oxarc, compressed air, 100 mL min^{-1}), or by treating a NH_4-ZSM-5 sample (Zeolyst, CBV3024E, Si/Al = 15) with $(NH_4)_2SiF_6$ using reported procedures to remove the extra-framework Al moieties, followed by calcination.[19]

Catalyst characterization. The Si and Al contents in the zeolite samples were measured by atomic absorption spectroscopy (AAS) on a UNICAM 939 AA–Spectrometer. The BET surface areas and pore volumes were determined by N_2 adsorption at 77 K using a Micromeritics ASAP 2020 system. Samples were degassed at 573 K for 5 h. The ultra-high field ^{27}Al MAS NMR experiments were performed on a Varian–Agilent Inova 63-mm wide-bore 850 MHz NMR spectrometer (magnetic field 19.97 T, Larmor frequency 221.4 MHz) with a commercial 3.2 mm pencil-type MAS probe. In a typical experiment, ~15 mg of fully hydrated sample powder were loaded in the rotor and measured at ambient temperature. A single pulse sequence with a pulse length of 2.0 ms, equivalent to a pulse angle of 45°, was selected for acquiring each spectrum with a recycle time of 1 s and total accumulation of 5000 scans. The spectra (Supplementary Fig. 13) were recorded at a spinning rate of 20 kHz ± 2 Hz and were referenced to 1.5 M aqueous solution of $Al(NO_3)_3$ (0 ppm) using the center of the octahedral peak of γ-Al_2O_3 (13.8 ppm) as a secondary reference.

Density functional theory. Periodic DFT calculations were carried out using the CP2K code.[23] All calculations employed a mixed Gaussian and planewave basis sets. The basis set superimposition error (BSSE) derived from Gaussian localized basis set used in our CP2K calculations has been estimated to be ~3 kJ/mol.[24] Core electrons were represented with Goedecker-Teter-Hutter pseudopotentials,[25] and the valence electron wavefunction was expanded in a double-zeta basis set with polarization functions along with an auxiliary plane wave basis set with an energy cutoff of 360 eV. The generalized gradient approximation exchange-correlation functional of Perdew, Burke, and Enzerhof[26] was used. Each reaction state configuration was optimized with the Broyden-Fletcher-Goldfarb-Shanno algorithm with SCF convergence criteria of 10^{-8} au. To compensate for the long-range van der Waals dispersion interaction between the adsorbate and the zeolite, the DFT-D3 scheme[27] was employed with an empirical damped potential term added into the energies obtained from exchange-correlation functional in all calculations. Important entropic contribution and zero-point energy (ZPE) corrections, which are important for zeolite-catalyzed reactions, have been taken into account.[28] More details, including the periodic structures of MFI and BEA used for calculations, can be found in our recent contributions.[2,3]

Error analysis for intrinsic activation parameters. The standard errors in $\Delta H^{\circ\ddagger}$ and $\Delta S^{\circ\ddagger}$ were determined from regression analysis of the dependence of the intrinsic rate constant on temperature via the rectified Eyring equation.

$$\ln\left(\frac{k_{rxn}}{T}\right) = \left(\ln\frac{k_B}{h} + \frac{\Delta S^{\ddagger}}{R}\right) - \frac{\Delta H^{\ddagger}}{R}\left(\frac{1}{T}\right) \tag{22}$$

The standard error in $\Delta G^{\circ\ddagger}$ was estimated from the quantities obtained from the sum of squares of residuals that is determined by the regression analysis of the intrinsic rate constant. Specifically, for zeolites, $TOF_z = k_z$, according to the Eyring equation,

$$\Delta G^{\circ\ddagger}=RT\ln(k_B/h)-RT\ln(TOF_z/T) \tag{23}$$

Here, $RT\ln(k_B/h)$ is a constant, so for $\Delta G^{\circ\ddagger}$, the only error source is $\Delta\ln(TOF_z/T)$. Then, we have $\Delta\Delta G^{\circ\ddagger}=RT*\Delta\ln(TOF_Z/T)$. The standard errors in $\Delta H^{\circ\ddagger}$ and $\Delta S^{\circ\ddagger}$ (Table 3 in the main text) are bigger due to the greater uncertainty in separating $\Delta G^{\circ\ddagger}$ into $\Delta H^{\circ\ddagger}$ and $\Delta S^{\circ\ddagger}$ as they are derived from the slope and intercept, respectively, of the Eyring plot.

Supplementary References

(1) Martínez, M., Verboekend, D., Pérez-Ramírez J. & Corma, A. Stabilized hierarchical USY zeolite catalysts for simultaneous increase in diesel and LPG olefinicity during catalytic cracking. *Catal. Sci. Technol.* **3**, 972–981 (2013).

(2) Liu, Y., Vjunov, A., Shi, H., Eckstein, S., Camaioni, D.M., Mei, D., Barath, E. & Lercher, J.A. Enhancing the catalytic activity of hydronium ions through constrained environments. *Nat. Commun.* **8**, 14113 (2017).

(3) Mei, D. & Lercher, J.A. Mechanistic insights into aqueous phase propanol dehydration in h-zsm-5 zeolite. *AIChE J.* **63**, 172-184 (2016).

(4) Holmes, J.L., McGillivray, D. & Rye, R.T.B. Specific and random processes in the fragmentation of cyclohexanol. *Org. Mass Spectrom.* **7**, 347–356 (1973).

(5) Thibblin, A. Mechanisms of solvolytic alkene-forming elimination reactions. *Chem. Soc. Rev.* **22**, 427–433 (1993).

(6) Ravenelle, R. M., Schüβler, F., D'Amico, A., Danilina, N., van Bokhoven, J. A., Lercher, J. A., Jones, C. W. & Sievers, C. Stability of Zeolites in Hot Liquid Water. *J. Phys. Chem. C* **114**, 19582–19595 (2010).

(7) Ennaert, T., Geboers, J., Gobechiya, E., Courtin, C. M., Kurttepeli, M., Houthoofd, K., Kirschhock, C. E. A., Magusin, P. C. M. M., Bals, S., Jacobs, P. A. & Sels, B. F. Conceptual Frame Rationalizing the Self-Stabilization of H-USY Zeolites in Hot Liquid Water. *ACS Catal.* **5**, 754–768 (2015).

(8) Vjunov, A., Derewinski, M. A., Fulton, J. L., Camaioni, D. M. & Lercher, J. A. Impact of Zeolite Aging in Hot Liquid Water on Activity for Acid-Catalyzed Dehydration of Alcohols. *J. Am. Chem. Soc.* **137**, 10374–10382 (2015).

(9) Wang, M., Xia, Y., Zhao, L., Song, C., Peng, L., Guo, X., Xue, N. & Ding, W. Remarkable acceleration of the fructose dehydration over the adjacent Brønsted acid sites contained in an MFI-type zeolite channel. *J. Catal.* **319**, 150–154 (2014).

(10) Zhang, L., Chen, K., Chen, B., White, J. L. & Resasco, D. E. Factors that Determine Zeolite Stability in Hot Liquid Water. *J. Am. Chem. Soc.* **137**, 11810–11819 (2015).

(11) Chen, K., Kelsey, J., White, J. L., Zhang, L. & Resasco, D. E. Water Interactions in Zeolite Catalysts and Their Hydrophobically Modified Analogues. *ACS Catal.* **5**, 7480–7487 (2015).

(12) Camblor, M. A., Corma, A., Iborra, S., Miquel, S., Primo, J. & Valencia, S. Beta Zeolite as a Catalyst for the Preparation of Alkyl Glucoside Surfactants: The Role of Crystal Size and Hydrophobicity. *J. Catal.* **172**, 76–84 (1997).

(13) Zapata, P. A., Faria, J., Ruiz, M. P., Jentoft, R. E. & Resasco, D. E. Hydrophobic Zeolites for Biofuel Upgrading Reactions at the Liquid–Liquid Interface in Water/Oil Emulsions. *J. Am. Chem. Soc.* **134**, 8570–8578 (2012).

(14) NIST database: http://webbook.nist.gov/cgi/cbook.cgi?ID=C108930&Mask=10#Solubility

(15) Staudinger, J.; Roberts, P.V. A critical compilation of Henry's law constant temperature dependence relations for organic compounds in dilute aqueous solutions. *Chemosphere* **44**, 561–576 (2001).

(16) Do, D. D. Pure Component Adsorption in Microporous Solids. In Adsorption Analysis: Equilibria and Kinetics; Imperial College Press: London, 1998; Vol. 2, pp 149–190.

(17) Lowry, T.H. & Richardson, K.S. Mechanism and Theory in Organic Chemistry, Addison Wesley Longman.

(18) Mellmer, M. A., Sener, C., Gallo, J. M. R., Luterbacher, J. S., Alonso, D. M. & Dumesic, J. A. Solvent Effects in Acid-Catalyzed Biomass Conversion Reactions. *Angew. Chem. Int. Ed.* **53**, 11872 –11875 (2014).

(19) Zhi, Y., Shi, H., Mu, L., Liu, Y., Mei, D., Camaioni, D. M. & Lercher, J. A. Dehydration Pathways of 1-Propanol on HZSM-5 in the Presence and Absence of Water. *J. Am. Chem. Soc.* **137**, 15781–15794 (2015).

(20) Gounder, R. Hydrophobic microporous and mesoporous oxides as Brønsted and Lewis acid catalysts for biomass conversion in liquid water. *Catal. Sci. Tech.* **4**, 2877–2886 (2014).

(21) Zapata, P. A., Huang, Y., Gonzalez-Borja, M. A. & Resasco, D. E. Silylated hydrophobic zeolites with enhanced tolerance to hot liquid water. *J. Catal.* **308**, 82–97 (2013).

(22) Prodinger, S., Derewinski, M. A., Vjunov, A., Burton, S. D., Arslan, I. & Lercher, J. A. Improving Stability of Zeolites in Aqueous Phase via Selective Removal of Structural Defects. *J. Am. Chem. Soc.* **138**, 4408–4415 (2016).

(23) Bandow, S. et al. Electronic and vibrational properties of Rb-intercalated MoS2 nanoparticles. *Mater. Sci. Eng. A* **204**, 222–226 (1995).

(24) VandeVondele, J. & Hutter, J. Gaussian basis sets for accurate calculations on molecular systems in gas and condensed phases. *J. Chem. Phys.* **127**, 114105 (2007).

(25) Goedecker, S., Teter, M. & Hutter, J. Separable dual-space Gaussian pseudopotentials. *Phys. Rev. B* **54**, 1703–1710 (1996).
(26) Perdew, J. P., Burke, K. & Ernzerhof, M. Generalized Gradient Approximation Made Simple. *Phys. Rev. Lett.* **77**, 3865–3868 (1996).
(27) Grimme, S., Antony, J., Ehrlich, S. & Krieg, H. A consistent and accurate ab initio parametrization of density functional dispersion correction (DFT-D) for the 94 elements H-Pu. *J. Chem. Phys.* **132**, 154104 (2010).
(28) De Moor, B. A. *et al.* Normal mode analysis in zeolites: toward an efficient calculation of adsorption entropies. *J. Chem. Theory Comput.* **7**, 1090–1101 (2011).
